# Electronic Effects of Substituents on *fac*-M(bpy-R)(CO)_3_ (M = Mn, Re) Complexes for Homogeneous CO_2_ Electroreduction

**DOI:** 10.3389/fchem.2019.00417

**Published:** 2019-06-05

**Authors:** Laura Rotundo, Emanuele Azzi, Annamaria Deagostino, Claudio Garino, Luca Nencini, Emanuele Priola, Pierluigi Quagliotto, Riccardo Rocca, Roberto Gobetto, Carlo Nervi

**Affiliations:** ^1^Department of Chemistry, Università degli Studi di Torino, Turin, Italy; ^2^NIS Interdepartmental Centre, Università degli Studi di Torino, Turin, Italy; ^3^Consorzio Interuniversitario Reattività Chimica e Catalisi (CIRCC), Bari, Italy

**Keywords:** CO_2_ electroreduction, Mn complexes, Re complexes, DFT calculations, bipy ligands, homogeneous catalysis, electron-withdrawing, electron-donating

## Abstract

Synthesis and characterization of 14 new 2,2′-bipyridine metal complexes *fac*-M(bpy-R)(CO)_3_X (where M = Mn, X = Br or M = Re, X = Cl and R = -CF_3_, -CN, -Ph, -PhOH, -NMe_2_) are reported. The complexes have been characterized by NMR, IR spectroscopy and elemental analysis. Single crystal X-Ray diffraction structures have been solved for Re(dpbpy)(CO)_3_Cl (dpbpy = 4,6-diphenyl-2,2′-bipyridine) and Re(hpbpy)(CO)_3_Cl (hpbpy = 4-(2-hydroxy-phenyl)-6-phenyl-2,2′-bipyridine). Electrochemical behaviors of the complexes in acetonitrile under Ar and their catalytic performances for CO_2_ reduction with added water and MeOH have been investigated by cyclic voltammetry and controlled potential electrolysis. The role of the substituents on the electrochemical properties and the related over potentials required for CO_2_ transformation have been analyzed. The complexes carrying only electron withdrawing groups like -CF_3_, -CN totally lose their catalytic activities toward CO_2_ reduction, whereas the symmetric -NMe_2_ substituted and push-pull systems (containing both -NMe_2_ and -CF_3_) still display electrocatalytic current enhancement under CO_2_ atmosphere. The complexes carrying a phenyl or a phenol group in position 4 show catalytic behaviors similar to those of simple M-bpy systems. The only detected reduction product by GC analysis is CO: for example, *fac*-Re (bpy-4,4′-NMe_2_)(CO)_3_Cl gives CO with high faradic efficiency and a TON of 18 and 31, in absence of external proton source and with 5% MeOH, respectively. DFT calculations were carried out to highlight the electronic properties of the complexes; results are in agreement with experimental electrochemical data.

## Introduction

Nowadays the CO_2_ concentration in the atmosphere is continuously increasing alongside with the overall world energy demand. Converting carbon dioxide via electrochemical reduction into useful chemicals and fuels for energy storage is an attractive and promising approach. CO_2_ reduction is a competition between its thermodynamic and kinetic: the one electron reduction in water occurs at a very negative potential (−1.90 V vs. SCE at pH 7) (Hammouche et al., 1988; Saveant, 2008) because it requires a drastic change in the geometry, from the linear CO_2_ molecule to the bent CO${}_{2}^{-•}$ radical anion. The reason of such high negative overpotential is due to the slow kinetics of the electron transfer, which is associated to the different geometries of the neutral and reduced species, respectively. However, the reduction reactions involving multiple electron transfers coupled with proton transfers provide a significant lowering of the thermodynamic barrier. To avoid the CO${}_{2}^{-•}$ as intermediate and to lower the energy cost of the reduction process, key catalytic strategies have been developed with the aim of obtaining the various products selectively. The best electrocatalysts currently studied work at a potential 100 mV negative with respect to E^0^ CO_2_/P (where P generically indicates the reduction products, CO, HCOOH, HCHO, CH_3_OH, CH_4_) (Francke et al., 2018; Franco et al., 2018). Despite the numerous advantages of heterogeneous electrocatalysis (Sun et al., 2016; Rotundo et al., 2019a), clever integration with the homogeneous counterpart allows a rational design of the catalysts, by tuning both the metal center and/or the ligand. One of the greatest challenge of the homogeneous approach lies in the search of stability, durability and improved turnover number (TON) efficiencies (Grice, 2017; Takeda et al., 2017; Wang et al., 2019). Bipyridine transition metal complexes represent one of the most studied classes of molecular electrocatalysts since the 1980s (Stanbury et al., 2018). Bipyridine has been extensively studied as ligand in the field of electro and photo-catalysis because of the capability to store electrons and subsequently delocalize electronic density on its π orbitals (Vlček, 2002; Elgrishi et al., 2017). Both *fac*-Re(bpy)(CO)_3_Cl and *fac*-Mn(bpy)(CO)_3_Br are capable of reducing CO_2_ to CO with high faradaic efficiency (Hawecker et al., 1984; Bourrez et al., 2011). Comparing these two complexes, Mn exhibits a catalytic peak that is shifted more anodically (around 300 mV, E_p_ = −1.51 V vs. Ag/AgCl in MeCN) in comparison with the second reduction of Re analogous (E_p_ = −1.8 V vs. Ag/AgCl in MeCN). Another important difference is that usually the Mn-bpy complexes show their catalytic activities only in the presence of external proton sources. To better shed light on this uniqueness, our research group synthesized Mn(pdbpy)(CO)_3_Br (Franco et al., 2014, 2017), in which two pendant phenolic groups act as local proton source (Costentin et al., 2012), capable of reducing CO_2_ even in anhydrous acetonitrile. In this case a considerable amount of HCOOH was also detected. Conversely the analogous complex in which the OH groups were replaced by methoxy groups did not show any catalytic activity without the addition of Brønsted acid.

The electrochemical behavior of the two well-known Re and Mn-bpy complexes could be reasonably altered by varying the bipyridine moiety, i.e., introducing electron-withdrawing and electron-donating groups (Machan et al., 2014; Walsh et al., 2015; Stanbury et al., 2018). Kubiak and his group investigated the effect of 4,4′-di-tertbutyl-2,2′-bipyridine (tBu_2_-bpy) firstly on rhenium carbonyl complexes, secondarily on manganese (Smieja and Kubiak, 2010; Smieja et al., 2013). In other works they studied the role of the modification in the 6,6′ position of the bipyridine (Sampson et al., 2014; Sampson and Kubiak, 2016). A similar approach has already been applied to Mo and W-bpy complexes (Franco et al., 2017; Rotundo et al., 2018), to both Mn and Re-bpy complexes by some of us (Franco et al., 2017; Rotundo et al., 2019b) and in the current work.

**Scheme 1:**
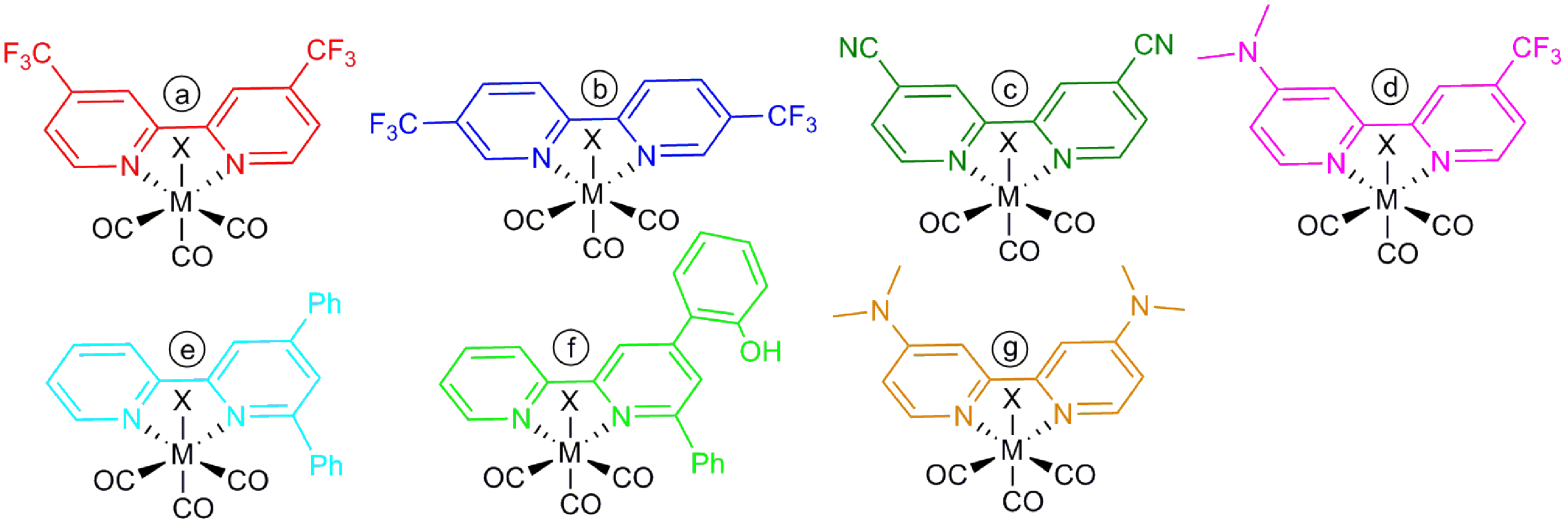
Chemical sketches of the investigated complexes, where M = Mn, X = Br, or M = Re, X = Cl.

The target of the modification is the reduction potential of the catalyst, namely $E_{cat}^{0}$: usually a less negative $E_{cat}^{0}$ corresponds to a decreased rate of CO_2_ conversion (Francke et al., 2018). Electronic properties of organic groups are commonly described by the inductive (±I) and mesomeric (±M) effects. Substituents with electron withdrawing groups like -CF_3_ (strong –I effect) and -CN (weaker –I effect) and with electron donating groups, like -N(Me)_2_ (strong +M effect), -Ph and -PhOH (weaker +M effect), were placed in the 4,4′, 4,6, and 5,5′ positions of the bipyridine ligand coordinated to the metals. Combining both push and pull effects in the so called “push-pull” system, an electronic gradient is forced through the bipyridine. In this paper we explore the electrocatalytic properties of novel Mn and Re bpy-type complexes, bearing 7 differently substituted ligands (Scheme 1). More generally electron-donating groups are expected to convey greater nucleophilicity to the metal-center, although catalysis should require higher overpotentials. DFT calculations have been used as complementary tool to better correlate experimental electrochemical data, whereas Controlled Potential Electrolysis (CPE) experiments are useful to elucidate the stability and durability of the catalysts in acetonitrile solutions.

## Results

### Synthesis and Structure

Fourteen new 2,2′-bipyridine metal complexes *fac*-M(bpy-R)(CO)_3_X where M = Mn, X = Br or M = Re, X = Cl are reported, namely Mn(bpy-4,4′-CF_3_)(CO)_3_Br (**1a**), Mn(bpy-5,5′-CF_3_)(CO)_3_Br (**1b**), Mn(bpy-4,4′-CN)(CO)_3_Br (**1c**), Mn(bpy-4,4′-CF_3_-NMe_2_)(CO)_3_Br (**1d**), Mn(dpbpy)(CO)_3_Br (**1e**), Mn(hpbpy)CO)_3_Br (**1f**), Mn(bpy-4,4′-NMe_2_)(CO)_3_Br (**1g**), Re(bpy-4,4′-CF_3_)(CO)_3_Cl (**2a**), Re(bpy-5,5′-CF_3_)(CO)_3_Cl (**2b**), Re(bpy-4,4′-CN)(CO)_3_Cl (**2c**), Re(bpy-4,4′-CF_3_-NMe_2_)(CO)_3_Cl (**2d**), Re(dpbpy)(CO)_3_Cl (**2e**), Re(hpbpy)(CO)_3_Cl (**2f**), Re(bpy-4,4′-NMe_2_)(CO)_3_Cl (**2g**).

The ligands and the corresponding Mn and Re complexes have been synthesized according to the procedure reported in the experimental section. The complexes have been characterized by NMR, IR spectroscopy and elemental analysis. Single crystal X-Ray diffraction structures have been solved for **2e** and **2f** (for XRD data see Tables S1–S6).

**Figure 1:**
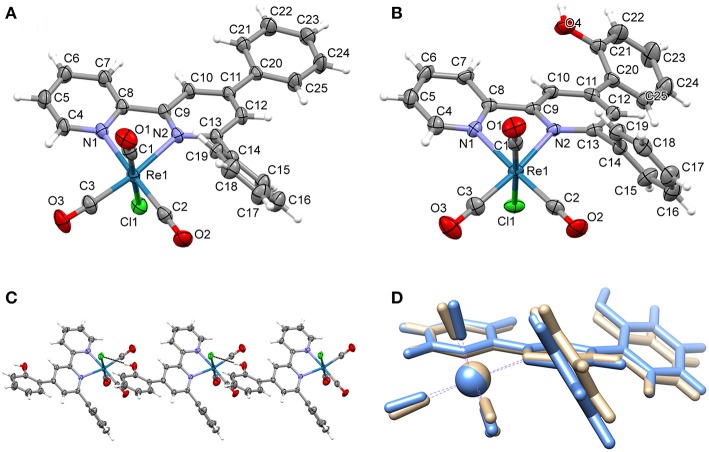
**(A)** molecular structure of Re(dpbpy)(CO)_3_Cl (**2e**, structure A), **(B)** molecular structure of Re(hpbpy)(CO)_3_Cl (**2f**), **(C)** chain formed by O–H···Cl intermolecular contacts in the crystal structure of **2f**, and **(D)** comparison of DFT optimized (light blue) and X-ray (brown) structures of **2f**.

The complex Re(dpbpy)(CO)_3_Cl (**2e**) crystallized from both acetonitrile and benzene solutions by slow evaporation, forming prismatic orange platelets of a phase of the pure molecular product (structure A, Figure S1a) and a solvate with two benzene molecules (structure B, Figure S1b). The first presents the monoclinic centrosymmetric P2_1_/a space group (Figure S1c) while the second the monoclinic centrosymmetric P2/n space group (Figure S1d). The complex Re(hpbpy)(CO)_3_Cl (**2f**) crystallized in dark by slow evaporation of solutions of toluene and benzene as yellow platelets and from ethyl acetate as orange prisms, both stable to air (Figure S2a). The crystal structure has been obtained from a platelet obtained from toluene solution and has monoclinic P2_1_/n space group type (Figure S2b). The structures of both **2e** and **2f** present a quite distorted octahedral geometry around the rhenium center, as can be seen by the values in Table 1. The coordination bond of N1 is 0.1 Å longer than that of N2 (see Figure 1 for numeration) and a similar asymmetry can be observed in the coordination of CO in trans position to the nitrogens. This asymmetric coordination, also present in other terpyridine derivatives (Anderson et al., 1990; Civitello et al., 1993; Wang et al., 2013; Klemens et al., 2016) is completely different from the very symmetrically bonded bpy derivatives (with N-Re average distances equal in the two coordinating pyridyl rings and long 2.17 Å). At the same time, while most of the 2,2′-bipyridine derivatives of *fac*-Re(CO)_3_Cl complex unit are almost planar respect to the basal OC–Re–CO plane (Kurz et al., 2006; Smieja and Kubiak, 2010; Bullock et al., 2012; Machan et al., 2014; Manbeck et al., 2015), in the case of **2e** and **2f**, the ligand is distorted outside this plane (see Table 1). This behavior can be detected in all the terpyridines, in which the third pyridine ring is not coordinated to the metal center and is equivalent to a phenyl ring, and in 6-phenyl substituted 2,2′-bipyridine derivatives. The reason of such distortion is clarified by considering the steric hindrance of the vicinal phenyl ring (the distance between the nearest CO group and the centroid of the phenyl ring in ortho to N1 is about 3.2 Å) that pushes up all the framework of the organic ligand, modifying the Re environment. This phenyl group is rotated to follow the shape of carbonyls with a torsion angle respect to the central pyridine ring of about 130°.

**Table 1:** Selected X-ray bond lengths [Å] and angles [°] for **2e** and **2f**.

	**2e (structure A)**	**2e (structure B)**	**2f**
Re–C1	1.906 (5)	1.919 (5)	1.919 (5)
Re–C2	1.936 (5)	1.952 (5)	1.935 (5)
Re–C3	1.890 (5)	1.933 (5)	1.898 (5)
Re–N1	2.160 (5)	2.177 (5)	2.164 (5)
Re–N2	2.220 (5)	2.233 (5)	2.214 (5)
N2–C13–C14–C15	132.85 (5)	125.66 (4)	125.47 (4)
C2O2–ph	3.110 (5)	3.239 (3)	3.233 (5)
Re1–N2–C11	165.28 (6)	162.38 (6)	159.09 (6)

The distortion effects observed in the solid state are predicted also by molecular DFT calculations. Indeed, the optimized geometries of **2e** and **2f** perfectly overlap with the experimentally determined structures (Figure 1D and Figure S3), confirming that these anomalies originate from the coordination of 6-phenyl substituted 2,2′-bipyridine ligands to carbonyl complexes and are not induced by crystal packing contributions.

The crystal packing of **2e** (both structure A and structure B) and **2f** is dominated by weak C–H···Cl, C–H···O and π···π stacking interactions. In the case of **2f**, the presence of -OH group on the organic ligand interacting with the chloride induces the formation of hydrogen-bonded molecular chains (see Figure 1C and Figure S2c). For a more detailed analysis on the crystal packing consults the Supplementary Material (see Figures S4–S9).

### Cyclic Voltammetry Under Ar

**Figure 2:**
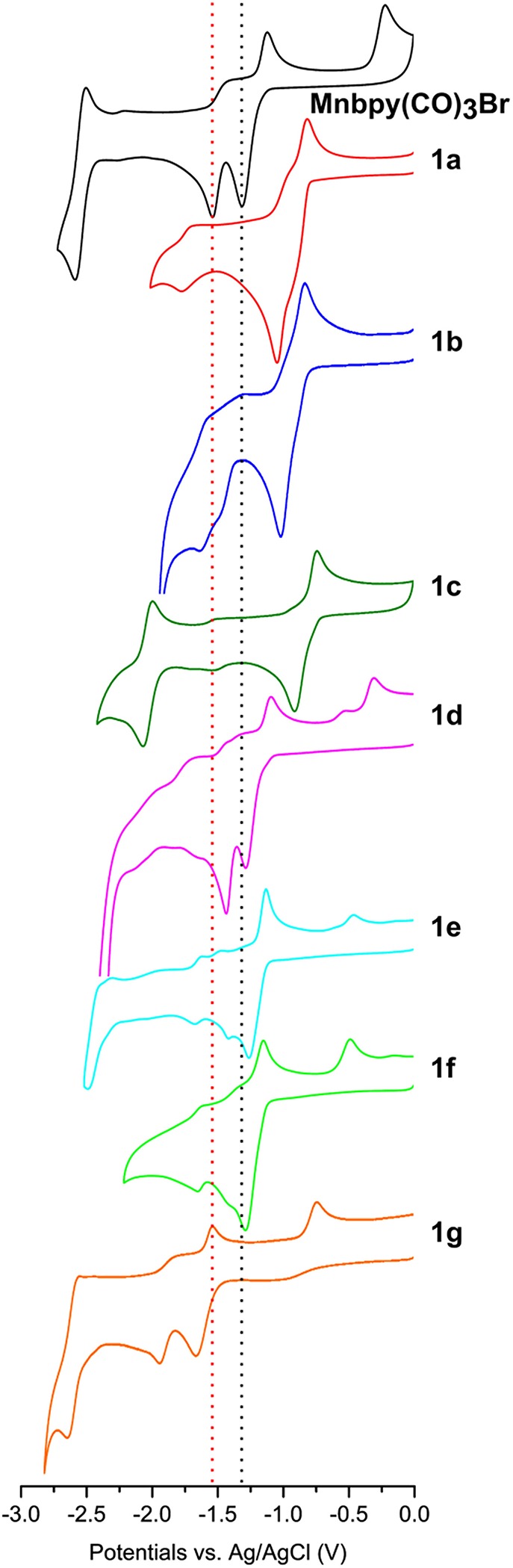
CVs of 1 mM solutions of **1a−1g** Mn complexes in MeCN/0.1 M TBAPF_6_ at GCE, scan rate 200 mVs^−1^ under Ar. CV of the reference *fac*-Mn(bpy)(CO)_3_Br is in black.

Cyclic Voltammetries (CVs) of all manganese complexes are reported in Figure 2. The CV of the Mn(bpy)(CO)_3_Br under our experimental conditions (in black) is included for comparison. This complex undergoes two successive irreversible reduction reactions and two reoxidation peaks (Bourrez et al., 2011). The first (E_p1_ = −1.29 V vs. Ag/AgCl) and the second (E_p2_ = −1.51 V vs. Ag/AgCl) reduction processes lead to the formation of the dimer and the mononuclear pentacoordinated anion species, respectively. Reoxidations of the pentacoordinated anion and of the dimer are located at −1.09 and −0.21 V vs. Ag/AgCl, respectively. The new synthesized complexes are supposed to display similar electrochemical behavior. Table 2 reports the peak potentials of the first and second reductions. As expected, the presence of –CF_3_ (**1a** and **1b**) and –CN (**1c**) shifts first and second potentials toward more positive values, when compared to Mn(bpy)(CO)_3_Br. In a recent paper (Rawat et al., 2019) DFT calculations suggested that electron-withdrawing substituents like -CF_3_ stabilize the radical anion. Furthermore, the formation of all Mn-Mn dimers was indicated as unfavorable. However, the electrochemical mechanism outlined above is commonly accepted, and the Mn dimer is strongly favored. We experimentally found that all complexes undergo a first and second chemically irreversible reductions. For example, **1a** shows two chemical irreversible processes followed by the reoxidations of the pentacoordinated radical anion and that of the dimer (Figure S10), even at high scan rates (1 V/s, Figure S11), thus confirming the general mechanism. CV of **1g** confirms that the strong electron-donating properties of dimethyl amino group result in more negative reduction potentials with respect to Mn(bpy)(CO)_3_Br. The push-pull system **1d**, **1e** and **1f** display similar potential values to the unsubstituted bpy-complex. In some complexes decomposition processes occurring after reduction generate small peaks (i.e., for **1e** and **1f** E_p_ = −1.65 and −1.63 V vs. Ag/AgCl are observed, respectively). The very negative peak around −3V vs. Ag/AgCl, is commonly assigned to a ligand-centered reduction.

**Table 2:** Reduction peak potentials [V] from the CV of Mn and Re complexes.

**Mn complexes**	**E_**p1**_**	**E_**p2**_**
Mn(bpy)(CO)_3_Br	−1.29	−1.51
**1a**	−0.92	−1.07
**1b**	−0.88	−0.99
**1c**	−0.89	−0.89
**1d**	−1.31	−1.42
**1e**	−1.26	−1.4
**1f**	−1.24	−1.39
**1g**	−1.64	−1.91
**Re complexes**	**E_1/2_ (or E_p1_)**	**E**_**p2**_
Re(bpy)(CO)_3_Cl	−1.35	−1.80
**2a**	−0.92	−1.45
**2b**	−0.83	−1.57
**2c**	−0.77	−1.85
**2d**	−1.29	−1.77
**2e**	−1.31	−1.68
**2f**	−1.37	−1.66
**2g**	−1.82 (1^st^ + 2^nd^ peak)	

**Figure 3:**
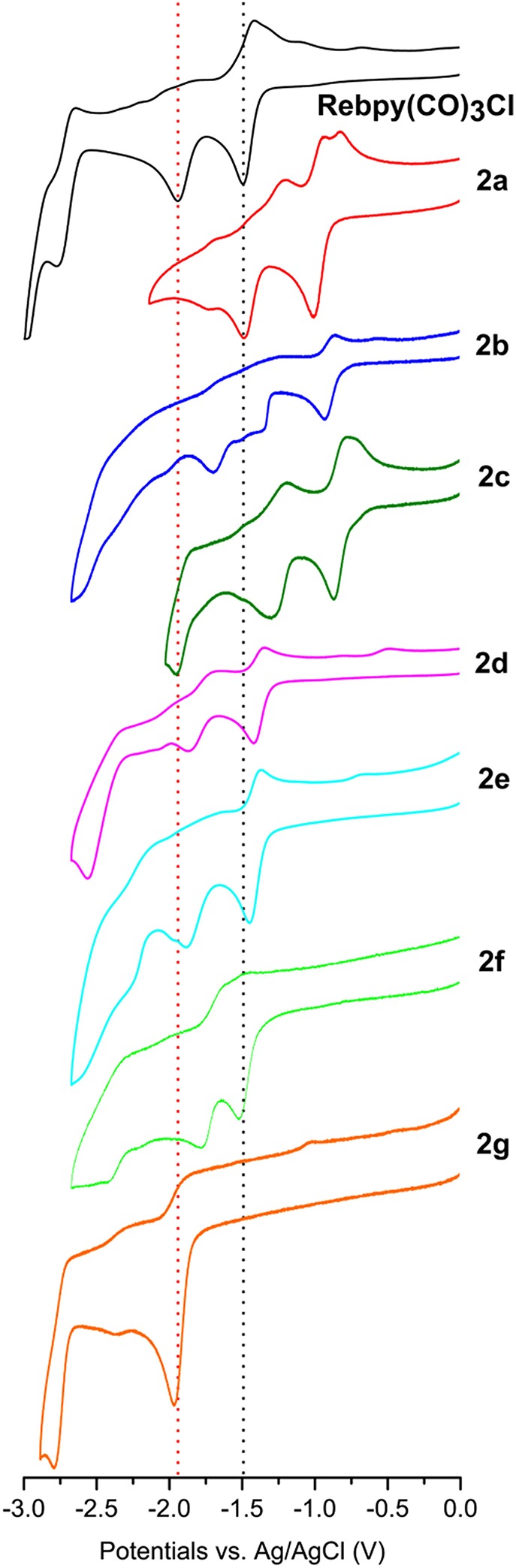
CVs of 1 mM solutions of **2a−2g** Re complexes in MeCN/0.1 M TBAPF_6_ at GCE, scan rate 200 mVs^−1^ under Ar. CV of the reference *fac*-Re(bpy)(CO)_3_Cl is in black.

CVs of all rhenium complexes are reported in Figure 3. CV of the reference Re(bpy)(CO)_3_Cl under our experimental conditions is included for comparison. This complex exhibits a first reversible reduction, due to the formation of the radical anion, which is more stable than the analogous with manganese, thus resulting in electrochemical reversibility and no presence of the reoxidation peak of the dimer (Hawecker et al., 1984). The second reduction leads to the pentacoordinated anion. Intrinsically, rhenium complexes require slightly higher overpotentials with respect to the corresponding manganese ones. The first reversible reduction of Re(bpy)(CO)_3_Cl is located at E_1/2_ = −1.35 V vs. Ag/AgCl, whereas the second chemically irreversible reduction is at E_p2_ = −1.80 V vs. Ag/AgCl. Similarly to manganese, the new synthesized rhenium complexes show no significant difference in the electrochemical pathway under Ar when compared to the reference Re(bpy)(CO)_3_Cl (Table 2). Electron withdrawing groups (**2a**, **2b**, and **2c**) shift the reduction processes toward less negative values; electron donating groups like in **2g** essentially merge the first and second reductions into a single peak. The push-pull system **2d**, in analogy with the case of **1d**, do not significantly alter the positioning of the reduction potentials. The third peak, around −2.5V, also for these complexes, is generally attributed to a bpy-centered reduction.

**Figure 4:**
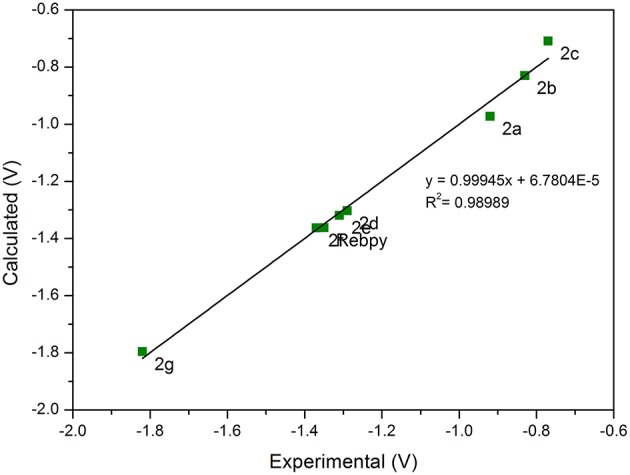
Plot of the calculated vs. experimental standard reduction potentials for **2a−2g**.

The reduction potentials estimated by DFT calculation are in excellent agreement with the experimental values obtained for the first reduction potential of all rhenium complexes (Figure 4). This confirms the reversible nature of the first reduction process, leading to stable radical anions characterized by a very small increase (about 0.05 Å) in the Re–Cl bond length (Figure S12). Conversely, in the case of manganese complexes, DFT calculations, which compute thermodynamic reduction potentials, are not suitable to estimate the irreversible electrochemical behavior of the compounds (Figure S13). Indeed, the high instability of the radical anion leads to the weakening of the Mn–Br bond, as clearly evidenced by the significant increase of the Mn–Br bond length in the anion structures (Figure S14). Our DFT calculations that include weak interactions, are in agreement with the chemically irreversibility of the first reduction and dimer production, even in the case of electron-withdrawing substituents. For example, the formation of the dimer [Mn(bpy)(CO)_3_]_2_ from its radical anion is favored by 143.5 kJ/mol, and in the case of **1a** is favored by 102.6 kJ/mol.

### Cyclic Voltammetry Under CO_2_

**Figure 5:**
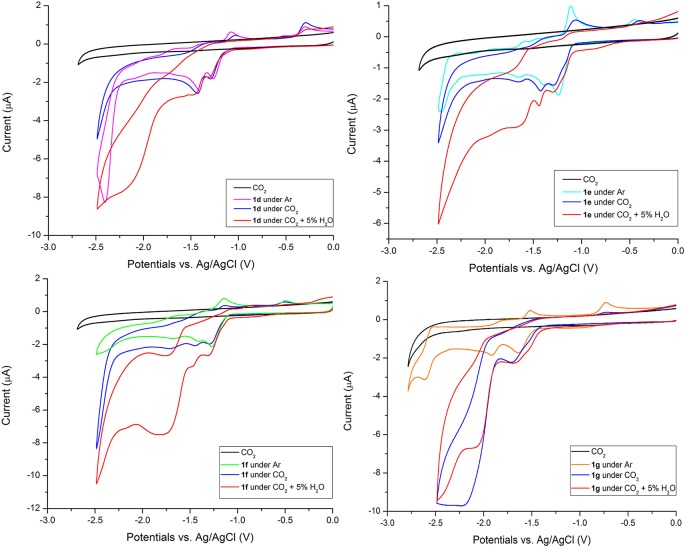
CVs of 0.5 mM solutions of **1d−1g** Mn complexes in MeCN/0.1 M TBAPF_6_ at GCE, scan rate 200 mVs^−1^ under Ar, under CO_2_ and with H_2_O (5%v). CV in black is the electrolyte saturated with CO_2_.

The electrochemical behavior of manganese complexes under CO_2_ and with H_2_O (5%v) is reported in Figure 5 for **1d** to **1g** and in Figure S15 for **1a**, **1b**, and **1c**, (these complexes are catalytically inactive toward CO_2_ reduction). CVs of **1a-1g** under CO_2_ with 5%v MeOH are included for comparison in Figure S15 too. All complexes do not show significant current increases switching from Ar to CO_2_ atmosphere. While this is expected for manganese complexes, **1g** shows a current increase, though limited, even in absence of Brønsted acid addition.

**Figure 6:**
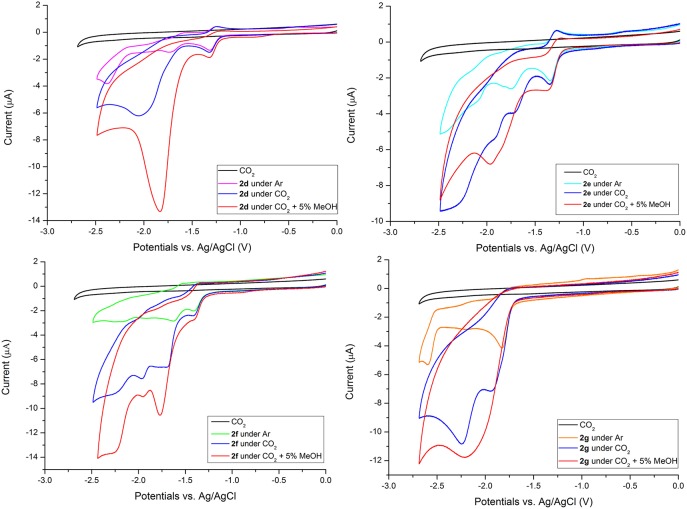
CVs of 0.5 mM solutions of **2d−2g** Re complexes in MeCN/0.1 M TBAPF_6_ at GCE, scan rate 200 mVs^−1^ under Ar, under CO_2_ and with 5%v MeOH. CV in black is the electrolyte saturated with CO_2_.

The electrochemical behavior of rhenium complexes under CO_2_ and with 5%v MeOH is reported in Figure 6 for **2d** to **2g** and in Figure S16 for **2a**, **2b**, and **2c**. CVs of **2a-2g** under CO_2_ with 5%v H_2_O are included for comparison in Figure S16 too. All complexes exhibit catalytic current switching from Ar to CO_2_ atmosphere, even in absence of Brønsted acid, as expected for rhenium complexes.

### Controlled Potential Electrolysis Experiments of the Complexes

Bulk electrolysis experiments of all manganese and rhenium complexes series under CO_2_ were performed upon setting the potential at values slightly negative to the second reductions, with and without external added Brønsted acids (water and methanol, 5%). A CO_2_ flow of 50 mL min^−1^ was kept constant during the experiments, gaseous products were determined by gas chromatography, and formate, if present, was assessed by NMR spectroscopy at the end of the experiments. Table 3 summarizes the results obtained during these CPE experiments. A general trend can be outlined: addition of water results in increased TONs for Mn complexes (Table 3 and Figure S17), differently from methanol, which drops them to lower values (Table S7). On the other hand, for the case of Re complexes, addition of methanol (Table 3 and Figure S18) seems to enhance the catalytic activity with respect to water, except for complex **2d**, which is catalytically inactive, despite the promising current increase in presence of MeOH under CO_2_.

**Table 3:** TON and faradic efficiencies (η) upon CPE (applied potential E in V vs. Ag/AgCl) of solutions of manganese and rhenium complexes (0.5 mM) in 0.1 M TBAPF_6_/MeCN in the presence of Brønsted acids (5%v).

**Complex**	**E [V]**	**T [min]**	**acid [5%]**	**TON_CO_**	**η_CO_ [%]**	**TON_H2_**	**η_H2_ [%]**
Mnbpy(CO)_3_Br	−1.6	240	H_2_O	13	100	–	–
**1d**	−1.5	420	H_2_O	26	84	1.7	16
**1e**	−1.5	300	H_2_O	12	72	2.3	10
**1f**	−1.5	300	H_2_O	13	64	3	20
**1g**	−1.95	120	–	3.5	85	–	–
**1g**	−1.95	300	H_2_O	13	90	–	–
Rebpy(CO)_3_Cl	−1.85	180	MeOH	15	80	–	–
**2d**	−1.8	180	MeOH	–	–	–	–
**2e**	−1.7	300	MeOH	5	56	–	–
**2f**	−1.7	240	–	5	64	–	–
**2f**	−1.7	300	MeOH	15	86	–	–
**2g**	−2	420	–	17.6	100	–	–
**2g**	−2	600	MeOH	31.5	100	–	–

## Conclusions

In summary, a systematic study of the effect of the electronic properties of the substituents on 2,2′-bipyridine Mn and Re complexes was conducted. Electron-withdrawing substituents shift the reduction potentials to more positive values, and eventually inhibit the catalytic activities of the corresponding Mn and Re complexes toward CO_2_ reduction. In the case of electron-donating substituents the opposite trend is observed. These observations are in agreement with the induced electron density localized on the metal, strongly influencing the change in the reactivity with the weak electrophile CO_2_. Increasing or decreasing the electron density on the metal should facilitate or prevent the formation of intermediate in which the CO_2_ is coordinated to the metal. Another interesting effect in varying the electron properties of the substituents is the merging of the first and second reduction processes, observed in some Re and Mn complexes. A judicious selection of bpy substituents provides an alternative way to the use of bulky substituents to prevent dimer formation (Sampson et al., 2014), with the aim of transforming two 1e reduction into a single **2e** reduction process (CO_2_ to CO reduction requires **2e**).

It is interesting to note that in spite of the presence of a -OH group in **1f**, no formic acid is detected, probably because in contrast with Mn carrying local proton sources (Franco et al., 2014, 2017) the hydroxyl group is located far from the metal center, thus the generation of the metal-hydride, commonly considered catalyst for formate production, is no longer entropically favored. It is interesting to note how CVs under CO_2_ of the push-pull systems **1d** and **2d** show enhanced catalytic currents; during CPE the Mn derivative **1d** displays the higher TON value, while the corresponding Re derivative **2d**, albeit from CV appears to be a potentially highly active catalyst, undergoes decomposition. While Mn catalysts suffer from the presence of MeOH, they appear to work better in water, which seems to react promptly with reduced Mn. In fact, even if the reduction potential of **1g** is rather negative (Table 2), no hydrogen is produced. This is in line with the high TON values observed for the CO_2_ electrochemical reduction in pure water by Mn electrocatalysts supported on electrode surface (Walsh et al., 2015; Reuillard et al., 2017; Rotundo et al., 2019b). DFT calculations performed with dispersion correction agree with the experimental data for both Mn and Re complexes. The Mn–Br bonds computed for the Mn radical anions undergo a significant elongation, around 0.2 Å, which indicate non-negligible weakening of the Mn–Br bonds. The release of halogen is also very probably favored by the polar and coordinating solvent MeCN; indeed Br substitution by MeCN has been experimentally observed not only in the radical anion, but also in neutral Mn species (Franco et al., 2017). We demonstrated here how the appropriate choice of the electron properties of the ligands is of critical importance in the design of more effective bipyridine Mn and Re electrocatalysts for CO_2_ reduction.

## Materials and Methods

### General Considerations

CV and CPE experiments were performed using a Metrohm Autolab 302n potentiostat. CO and H_2_ as CO_2_ reduction products were detected and quantified by an Agilent 490 Micro GC. NMR spectra were recorded on a JEOL ECP 400 FT-NMR spectrometer (^1^H operating frequency 400 MHz) or on a JEOL ECZR 600 FT-NMR spectrometer (^1^H operating frequency 600 MHz) at 298 K. ^1^H and ^13^C chemical shifts are reported relative to TMS (δ = 0) and referenced against solvent residual peaks. IR-ATR spectra were collected on a Fourier transform Equinox 55 (Bruker) spectrophotometer equipped with an ATR device; resolution was set at 2 cm^−1^ for all spectra. A spectral range of 400–4,000 cm^−1^ was scanned, using KBr as a beam splitter. GC-MS spectra were obtained on a mass selective detector Agilent 5,970 B operating at an ionizing voltage of 70 eV connected to a HP 5,890 GC equipped with a HP-1 MS capillary column (25 m length, 0.25 mm I.D., 0.33 μm film thickness. Elemental analyses (C, H, N) were performed on a Fisons Instruments EA-1108 CHNS-O Elemental Analyzer. ESI-MS spectra were recorded with a Thermo Advantage Max Spectrometer equipped with an ion trap analyzer and an ESI ion source.

### Synthesis and Characterization of Ligands

Flasks and all equipment used for the generation and reaction of moisture-sensitive compounds were dried by electric heat gun under N_2_. All commercially obtained reagents and solvents were used as received. Anhydrous DMF was purchased by Sigma Aldrich. Products were purified by preparative column chromatography on Macherey Nagel silica-gel for flash chromatography, 0.04–0.063 mm/230–400 mesh. Reactions were monitored by TLC using silica-gel on TLC-PET foils Fluka, 2–25 μm, layer thickness 0.2 mm, medium pore diameter 60 Å. hpbpy (4-(2-hydroxy-phenyl)-6-phenyl-2,2′-bipyridine) was synthesized by using the Kröhnke (1976) reaction and subsequently coupling the pyridinium iodide salt (**1**) with the corresponding hydroxy chalcone (**2**). dpbpy (4,6-diphenyl-2,2'-bipyridine) was synthesized according to reported procedure (Franco et al., 2015).

*Synthesis of 4,4*′*-bis(trifluoromethyl)-2,2*′*-bipyridine*: the method of O'Donnell et al. (2016) was slightly modified to synthesize 4,4′-bis(trifluoromethyl)-2,2′-bipyridine first reported by Furue et al. (1992). In a degassed 20 mL screw cap vial a solution of 2-bromo-4-(trifluoromethyl)pyridine (500 mg, 2.2 mmol) in 7.5 mL of anhydrous DMF was poured and degassed for 5 min. Pd(OAc)_2_ (25 mg, 0.11 mmol) was added later and the mixture degassed for additional 5 min. TBAI (815 mg 2.2 mmol), anhydrous K_2_CO_3_ (460 mg, 3.3 mmol), and i-PrOH (0.35 mL, 4.4 mmol) were then added to the mixture, subsequently heated at 100°C for 20 h. The heating was suspended, and the reaction mixture was filtered through a pad of celite. The filtrate was diluted in 25 mL of DCM and was washed with deionized water (3 × 25 mL). The organic phase was collected, and the water layer further extracted with 10 mL of DCM. The collected organic phase was dried with anhydrous MgSO_4_, filtered, and the solvent removed under reduced pressure to afford the crude solid that was purified by column chromatography on silica gel (eluent PE/AcOEt 10/1) to afford 220 mg of white solid as product. (Yield = 80%).

*Synthesis of [2,2*′*-bipyridine]-4,4*′*-dicarbonitrile*: the procedure reported by Losse et al. (2008) was followed with slight modifications. 4-Cyanopyridine (700 mg, 6.7 mmol) and 10% Pd/C (50 mg) were added into a 25 mL round-bottomed flask and five vacuum/nitrogen cycle were operated to minimize the amount of oxygen, then the flask was connected to a reflux condenser under nitrogen atmosphere. The mixture was heated to 230°C in a sand bath in order to reflux the 4-Cyanopyridine. After 24 h the mixture was cooled to room temperature, CHCl_3_ (15 mL) was added and the black suspension filtered through a frit. Solvent was removed under reduced pressure from the pale-yellow solution obtained until the product started to crystallize. Pentane (15 mL) was then added, and the concentrated solution cooled at 4–6°C for 16 h. The precipitate was filtered on Buchner funnel and washed with cold EtOH and dried to yield 100 mg of the product as a yellow-orange solid. (Yield 15%).

*General procedures for synthesis of 2-substitued N,N-dimethylpyridin-4-amine derivatives*. The previously reported procedure of Cuperly et al. (2002) was adapted according to the used electrophile. 2-(dimethylamino)-ethanol (0.8 mL, 8.0 mmol) was added to a three-necked round bottomed flask and dissolved in hexane (10 mL) under a N_2_ atmosphere. The solution was cooled at −5°C, BuLi (2.5 M, 6.4 mL, 16.0 mmol) was added dropwise for 10 min and the resulting mixture stirred for 40 min at −5°C. 4-DMAP (488 mg, 4.0 mmol) was then added and stirring continued for additional 60 min at 0°C, then the reaction medium was cooled at −78°C and the solution of the appropriate electrophile (10.0 mmol) was added dropwise by mean of a dropping funnel with pressure balance. Once the addition of the electrophile was completed the temperature was allowed to raise to 0°C (1.5 h) and the reaction quenched with deionized water at this temperature.

*Synthesis of N,N-dimethyl-2-(tributylstannyl)pyridin-4-amine*. 2-(dimethylamino)ethanol (0.8 mL, 8.0 mmol) in hexane (10 mL), BuLi (2.5 M, 6.4 mL, 16.0 mmol), and 4-DMAP (0.488 g, 4.0 mmol) were reacted as previously described then a solution of Bu_3_SnCl (3.255 g, 10.0 mmol) in 15 mL of hexane was added dropwise for 20 min. The reaction was quenched with deionized water (15 mL) then the aqueous phase was extracted with DCM (2 × 20 mL) and AcOEt (2 × 20 mL). The collected organic phase was dried with anhydrous Na_2_SO_4_, filtered, and the solvent removed under reduced pressure to afford a crude orange oil, that was used without further purification in the cross-coupling reactions (NMR-calculated yield = 69%).

*Synthesis of 2-iodo-N,N-dimethylpyridin-4-amine*: 2-(dimethylamino)ethanol (1.6 mL, 16.0 mmol) in hexane (25 mL), BuLi (2.5 M, 12.8 mL, 32.0 mmol), and 4-DMAP (0.980 g, 8.0 mmol) were reacted as previously described, then a solution of resublimed I_2_ (5.080 g, 20.0 mmol) in 50 mL of freshly distilled Et_2_O was added dropwise for 35 min. The reaction was quenched with a saturated solution of Na_2_S_2_O_3_ (25 mL) and stirred for additional 20 min at 0°C. The organic phase was separated and washed again with Na_2_S_2_O_3_ solution (2 × 15 mL) and brine (2 × 15 mL). The collected organic phase was dried with anhydrous Na_2_SO_4_, filtered, and the solvent removed under reduced pressure to afford a crude brown solid that was purified by column chromatography on silica gel (eluent: PE/AcOEt 5/5) to afford 1.683 g of white solid as product (Yield = 86%).

*General Procedure for Stille cross-coupling of N,N-dimethyl-2-(tributylstannyl)pyridin-4-amine with 2-halopyridine*. In a degassed 50 ml screw cap vial freshly distilled toluene (25 mL) was added and degassed for 10 min then Pd(OAc)_2_ (22 mg, 0.1 mmol) and PPh_3_ (52 mg, 0.2 mmol) were added in one portion. The resulting mixture was stirred and degassed until toning of the solution to red. Subsequently, *N,N*-dimethyl-2-(tributylstannyl)pyridin-4-amine (370 mg, 0.9 mmol), LiI (40 mg, 0.3 mmol), and CuI (57 mg, 0.3 mmol) were added to the mixture, then degassed for additional 5 min. Finally, the appropriate 2-halopyridine (1.1 mmol) was poured in the reaction medium that was kept under N_2_ atmosphere and heated to reflux for 16 h. After being cooled at room temperature, the resulting mixture was diluted with EtOAc (30 mL) and washed with a NH_4_OH solution (10 M) until the water layer did not turn blue any more, indicating that all the copper had been extracted. The collected organic phase was filtered on a pad of celite, diluted with DCM (30 mL) and dried with anhydrous Na_2_SO_4_. Then the solvents were removed under reduced pressure to afford the crude solid subsequently purified to yield the desired product.

*Synthesis of N*^4^*,N*^4^*,N*^4^'*,N*^4^'*-tetramethyl-[2,2*′*-bipyridine]-4,4*′*-diamine*: Following the general procedure, *N,N*-dimethyl-2-(tributylstannyl)pyridin-4-amine (370 mg, 0.9 mmol) and 2-iodo-*N*,*N*-dimethylpyridin-4-amine (270 mg, 1.1 mmol) in 25 mL of freshly distilled toluene were reacted to afford a crude yellowish solid that was suspended in 25 mL of Et_2_O and vigorously stirred for 30 min. The desired product was separated by filtration to yield 115 mg of a brown powder (Yield = 53 %).

*Synthesis of N,N-dimethyl-4*′*-(trifluoromethyl)-[2,2*′*-bipyridin]-4-amine:* following the general procedure, *N,N*-dimethyl-2-(tributylstannyl)pyridin-4-amine (370 mg, 0.9 mmol) and commercially available 2-bromo-4-(trifluoromethyl)pyridine (250 mg, 1.1 mmol) in 25 mL of freshly distilled toluene were reacted to afford a crude brown solid that was purified by column chromatography on silica gel (eluent: MeOH/DCM 5/95) to yield 169 mg of the desired product as a brown powder (Yield = 70%).

*Synthesis of N-(2-pyridylacetyl)-pyridinium iodide (****1****):* in a three-necked flask 82.5 mmol of 2-acetylpyridine and 90.5 mmol of iodine are dissolved in 100 ml of pyridine. The solution is refluxed for 3 h. The shiny black precipitate is then filtered and washed with diethyl ether (20 ml). The solid is recrystallized in hot ethanol: fine-scaled golden crystals are obtained (overall yield: 54%).

*Synthesis of (E)-3-(2-hydroxyphenyl)-1-phenyl-2-propen-1-one (****2****):* is synthesized by Claisen-Schmidt condensation of salicylaldehyde (50 mmol) with acetophenone (40 mmol) dissolved in 50 mL of ethanol (Tatsuzaki et al., 2006; Yin et al., 2012). The solution is vigorously stirred and 10 mL of KOH 40% (w/w) is dropwise added. The mixture is then heated to 60°C for 2–4 h until the disappearance of the starting reagents, monitored by TLC (EtAc: hexane 1:4). Once the reaction is completed, the suspension is poured into cold distilled water and acidified with HCl 2 M (final pH 2–3). The resulting precipitate is collected and washed once with water and then with cyclohexane (yield: 70%).

*Synthesis of 4-(2-hydroxy-phenyl)-6-phenyl-2,2*′*-bipyridine* (hpbpy): precursors **1** (4.8 mmol) and **2** (4.8 mmol) are added to a three-necked flask with an excess of ammonium acetate (48 mmol) and glacial acetic acid (4 mL). The solution is refluxed for 3 h. The glacial acetic acid is removed, and the residue dissolved in methanol. The yellowish product crystallizes on cooling.

*Synthesis of 4,6-diphenyl-2,2*′*-bipyridine* (dpbpy): is already reported in literature; NMR data are in agreement with previously published data (Cave and Raston, 2001).

### Synthesis of Mn Complexes

[Mn(CO)_5_Br] (0.100 mmol, 1 equiv) and the corresponding bipyridyl ligand (0.101 mmol, 1,01 equiv) were dissolved in sealed flasks containing 5 mL of diethyl ether (anhydrous for complex **1f**), and heated in a Biotage Initiator^+^ microwave reactor. When the microwave vial has been inserted into the microwave cavity and the cavity lid has been closed, high-frequency microwaves (2.45 GHz), generated by the magnetron, heat the reaction mixture at a constant temperature of 75°C for 15 min. The reaction mixtures were cooled to room temperature and the products centrifuged and washed once with diethyl ether. Yields of reactions: (**1a:** 75%, **1b:** 84%**, 1c:** 70%**, 1d**: 81%, **1e:** 68%, **1f:** 65%, **1g:** 78%).

### Synthesis of Re Complexes

[Re(CO)_5_Cl] (0.100 mmol, 1 equiv) and the corresponding bipyridyl ligand (0.101 mmol, 1.01 equiv) were dissolved in sealed flasks containing 5 mL of toluene (anhydrous for complex **2f**), and heated in the Biotage microwave reactor at a constant temperature of 130°C for an hour. After cooling of the reaction mixtures to room temperature, petroleum ether was added to precipitate the products, which were then centrifuged, filtered and washed once with cold diethyl ether. Yields of reactions: (**2a:** 70%, **2b:** 82%**, 2c:** 67%**, 2d**: 85%, **2e:** 65%, **2f:** 65%, **2g:** 75%).

### Elemental Analysis of the Complexes

The samples for microanalyses were dried in vacuum to constant weight (20°C, ca. 0.1 Torr). Elemental analysis (C, H, N) was performed in-house with a Fisons Instruments 1108 CHNS-O Elemental Analyzer.

Anal. Calcd.(%) for **1a (C**_**15**_**H**_**6**_**BrF**_**6**_**N**_**2**_**O**_**3**_**Mn)**: C 35.25; H, 1.18; N, 5.48. Found: C 35.49, H 1.25, N, 5.36.

Anal. Calcd.(%) for **1b (C**_**15**_**H**_**6**_**BrF**_**6**_**N**_**2**_**O**_**3**_**Mn)**: C, 35.25; H, 1.18; N, 5.48. Found: C 35.55, H 1.09, N 5.57.

Anal. Calcd.(%) for **1c (C**_**15**_**H**_**6**_**BrN**_**4**_**O**_**3**_**Mn)**: C, 42.38; H, 1.42; N, 13.18. Found: C 42.62, H, 1.50, N, 13.07.

Anal. Calcd.(%) for **1d (C**_**16**_**H**_**12**_**BrF**_**3**_**N**_**3**_**O**_**3**_**Mn)**: C, 39.53; H, 2.49; N, 8.64. Found: C, 39.41, H, 2.61, N, 8.75.

Anal. Calcd.(%) for **1e (C**_**25**_**H**_**16**_**BrN**_**2**_**O**_**3**_**Mn)**: C, 56.95; H, 3.06; N, 5.31. Found: C, 57.13, H, 2.96, N, 5.45.

Anal. Calcd.(%) for **1f (C**_**25**_**H**_**16**_**BrN**_**2**_**O**_**4**_**Mn)**: C, 55.27; H, 2.97; N, 5.16. Found: C, 55.15, H, 3.14, N, 4.95.

Anal. Calcd.(%) for **1g (C**_**17**_**H**_**17**_**BrN**_**4**_**O**_**3**_**Mn)**: C, 44.37; H, 3.72; N, 12.17. Found: C, 44.51, H, 3.82, N, 12.04

Anal. Calcd.(%) for **2a (C**_**15**_**H**_**6**_**ClF**_**6**_**N**_**2**_**O**_**3**_**Re)**: C, 30.13; H, 1.01, N, 4.69. Found: C, 30.20, H, 1.12, N, 4.35.

Anal. Calcd.(%) for **2b (C**_**15**_**H**_**6**_**ClF**_**6**_**N**_**2**_**O**_**3**_**Re)**: C, 30.13; H, 1.01, N, 4.69. Found: C, 29.95, H, 1.16, N, 4.78.

Anal. Calcd.(%) for **2c (C**_**15**_**H**_**6**_**ClN**_**4**_**O**_**3**_**Re)**: C, 35.20; H, 1.18; N, 10.95. Found: C, 35.34, H, 1.26, N, 10.77.

Anal. Calcd.(%) for **2d (C**_**16**_**H**_**12**_**ClF**_**3**_**N**_**3**_**O**_**3**_**Re)**: C, 33.54; H, 2.11; N, 7.33. Found: C, 33.43, H, 2.26, N, 7.48.

Anal. Calcd.(%) for **2e (C**_**25**_**H**_**16**_**ClN**_**2**_**O**_**3**_**Re)**: C, 48.90; H, 2.63; N, 4.56. Found: C, 49.06, H, 2.75, N, 4.43.

Anal. Calcd.(%) for **2f (C**_**25**_**H**_**16**_**ClN**_**2**_**O**_**4**_**Re)**: C, 47.66; H, 2.56; N, 4.45. Found: C, 47.53, H, 2.78, N, 4.36.

Anal. Calcd.(%) for **2g (C**_**17**_**H**_**17**_**ClN**_**4**_**O**_**3**_**Re)**: C, 37.33; H, 3.13; N, 10.24. Found: C, 37.25, H, 3.22, N, 10.16.

### Single-Crystal X-Ray Diffraction

The single-crystal data were collected with a Gemini R Ultra diffractometer with graphite-monochromated Mo-Kα radiation (λ = 0.71073) by the ω-scan method. The cell parameters were retrieved with the CrysAlisPro (Agilent, 2015) software, and the same program was used to perform data reduction with corrections for Lorenz and polarizing effects. Scaling and absorption corrections were applied through the CrysAlisPro1 multiscan technique. The structures of complex **2e** (both structure A and B) were solved with direct methods, while in the case of **2f** a meaningful initial guess for electron density was obtained only with Patterson Function by using SHELXS-14 (Sheldrick, 2008, 2015). All the structures were refined with full-matrix least-squares techniques on F^2^ with SHELXL-14 (Macrae et al., 2006) using the program Olex2 (Dolomanov et al., 2009). All non-hydrogen atoms were refined anisotropically. Hydrogen atoms were calculated and riding on the corresponding bonded atoms. The graphic of the crystal structures was generated using Mercury 3.9 (Macrae et al., 2006). CCDC codes 1891407–1891409 contain the supplementary crystallographic data for **2e** (structure A), **2e** (structure B) and **2f**. These data can be obtained free of charge via https://www.ccdc.cam.ac.uk/conts/retrieving.html, or from the Cambridge Crystallographic Data Center, 12 Union Road, Cambridge CB2 1EZ, UK; fax: (+44) 1223-336-033; or e-mail: deposit@ccdc.cam.ac.uk.

### CV and CPE Experiments

Acetonitrile used for the experiments was freshly distilled over calcium hydride and purged with Ar before use. 0.5–1 mM solutions of the complexes were prepared with tetrabutylammonium hexafluorophosphate (TBAPF_6_, Sigma-Aldrich, 98%) as supporting electrolyte (0.1 M). A single-compartment cell was employed for CV measurements, equipped with working a glassy carbon electrode (GCE, Ø = 1 mm), alongside a Pt counter electrode and a Ag/AgCl (KCl 3 M) reference electrode. The Ar- and CO_2_-saturated conditions were achieved by purging gases for 5 min before each potential sweep. A double-compartment H-type cell was used for CPE measurements, thus allowing to separate through a glass frit the anodic compartment from the cathodic one (a Pt wire is placed as counter electrode). A glassy carbon rod is employed as working electrode jointly with the Ag/AgCl reference electrode. A controlled and constant flow of CO_2_ (50 mL min^−1^) was maintained during the CPE measurements by means of a Smart Trak 100 (Sierra) flow controller. Under these experimental conditions, the redox couple (Fc^+^/Fc) is located at E_1/2_ = 0.35 V.

### Quantitative Analysis of CO_2_ Reduction Products

μGC measurements were used to detect CO and H_2_. Two modules equipped with CP-Molsieve 5 Å columns were kept at 105°C and at a pressure of 30 and 28 psi, with a thermal conductivity detector. The carrier gases were Ar for H_2_ and He for CO detection, respectively. The backflush vent option time was set to 7 s. The gas inside the measurement cell was sampled for 30 s every 3 min to fill the Micro GC 10 μL sample loop, and eventually 500 nL was injected into the column for the analyses. Instrument calibration was carried out measuring two different certified standards of CO and H_2_ in Ar matrix (Rivoira). Formate production was assessed by NMR spectroscopy.

### Computational Details

All the calculations were performed by the Gaussian 16 Revision B.01 (G16) program package (Frisch et al., 2016), employing density functional theory (DFT). Calculations were run using the Becke three-parameter hybrid functional (Becke, 1993), and the Lee–Yang–Parr gradient-corrected correlation functional (B3LYP) (Lee et al., 1988). Dispersion effects were added as semiempirical corrections with Becke–Johnson damping approach (GD3BJ) (Grimme et al., 2010, 2011). The solvent effect was included using the conductor-like polarizable continuum model (CPCM) with acetonitrile as solvent (Miertus et al., 1981). The def2TZVP basis set and effective core potential were used for the Mn, Br, and Cl atoms and the def2-SVP basis set was used for all the other atoms (Weigend and Ahlrichs, 2005). Unrestricted open-shell calculations were performed on the radical anions. Geometry optimizations were carried out without any symmetry constraints. The nature of the stationary points in the potential energy hypersurface was characterized by using harmonic vibrational frequency calculations. No imaginary frequencies were found, thus indicating we had located the minima on the potential-energy surfaces. Molecular-graphic images were produced by using the UCSF Chimera package from the Resource for Biocomputing, Visualization, and Informatics at the University of California, San Francisco (Pettersen et al., 2004).

## Author Contributions

RG and CN as corresponding authors wrote and revised the manuscript. EP performed Crystal X-ray structures. EA, AD, and PQ made the synthesis of the ligands. LR, RR, and LN synthesized the organometallic catalysts and performed the electrochemical and GC measurements. CG and CN made the DFT calculations.

### Conflict of Interest Statement

The authors declare that the research was conducted in the absence of any commercial or financial relationships that could be construed as a potential conflict of interest.
