# Supplementary material for: Electronic Effects of Substituents on fac-M(bpy-R)(CO)3 (M = Mn, Re) Complexes for Homogeneous CO2 Electroreduction
Source: Front Chem. 2019 Jun 5;7:417. doi: 10.3389/fchem.2019.00417 (PMC6561311; doi:10.3389/fchem.2019.00417)
Supplement: Supplementary file 1 [file Table_1.docx]

**SUPPLEMENTARY MATERIAL**

**Electronic Effects of Substituents on *fac*-M(bpy-R)(CO)_3_ (M = Mn, Re) Complexes for Homogeneous CO_2_ Electroreduction**

L. Rotundo^1,2,3^, E. Azzi^1^, A. Deagostino^1^, C. Garino^1,2,3^, L. Nencini^1^, E. Priola^1,2^, P. Quagliotto^1,2^, R. Rocca^1,2,3^, R. Gobetto^1,2,3^*, C. Nervi^1,2,3^*

^1^ Department of Chemistry, Università degli Studi di Torino, Torino, Italy, ^2^ NIS Interdepartmental Centre, Università degli Studi di Torino, Torino, Italy, ^3^ Consorzio Interuniversitario Reattivitá Chimica e Catalisi (CIRCC), Bari, Italy

| (a) | (b) |
| --- | --- |
| 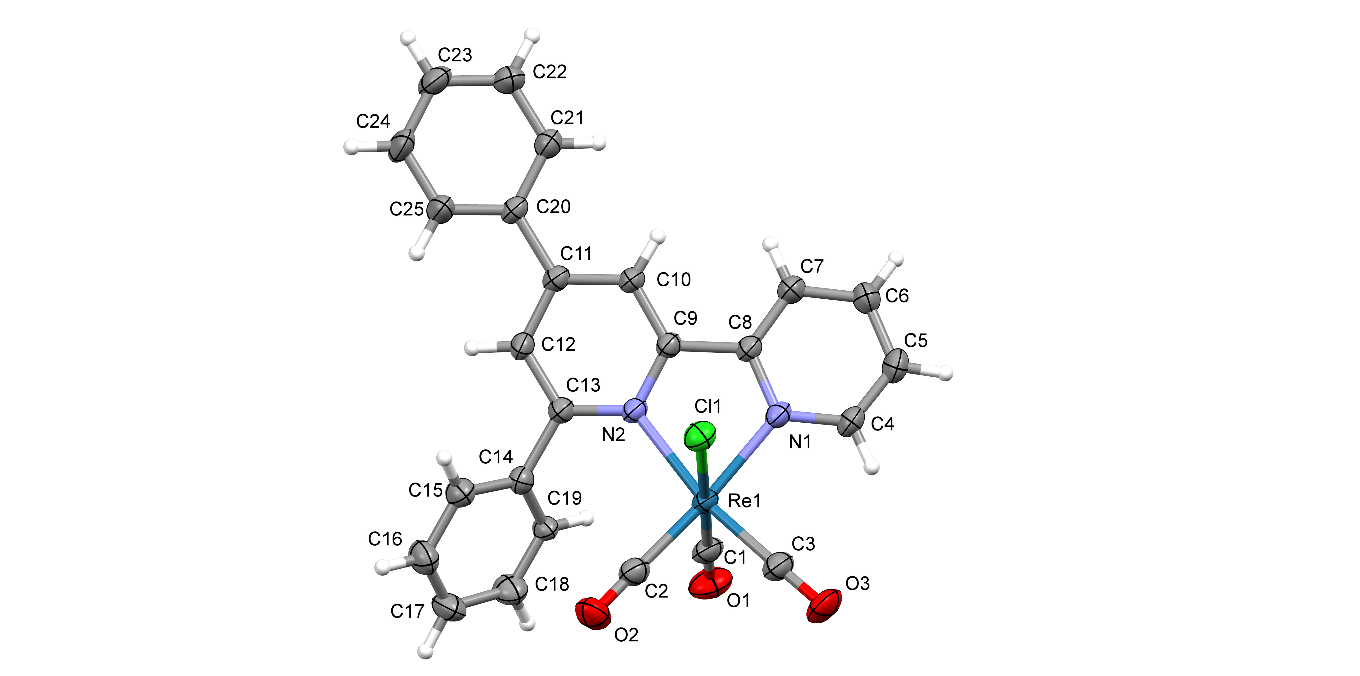 | **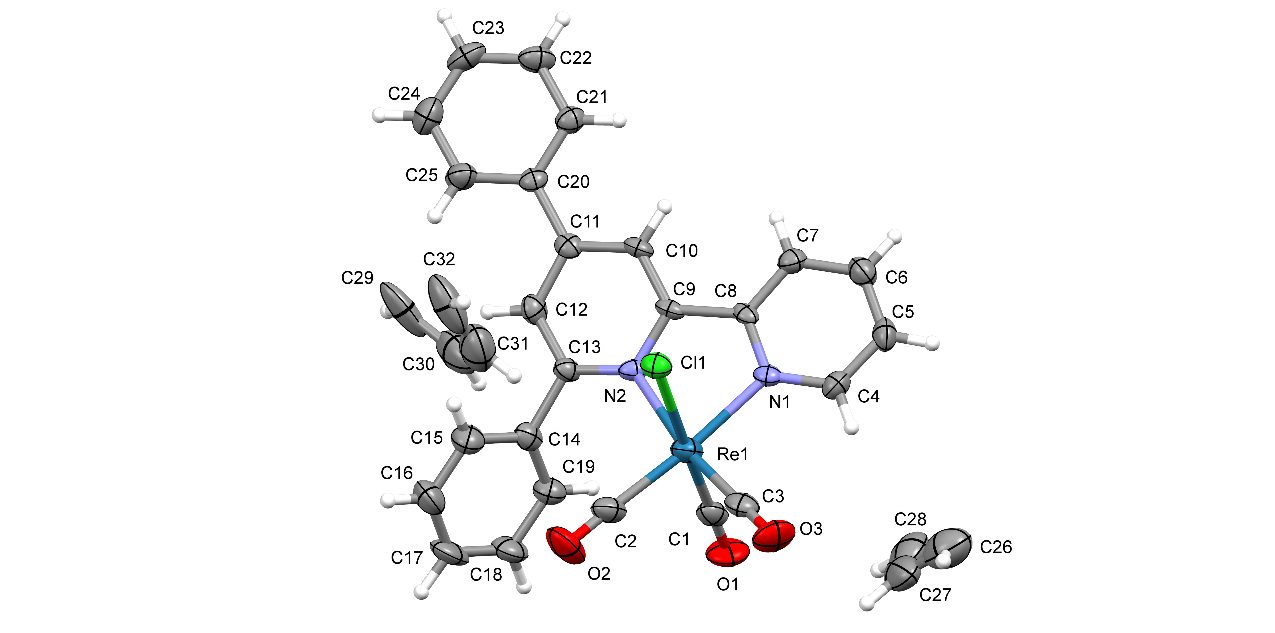** |
| (c) | (d) |
| 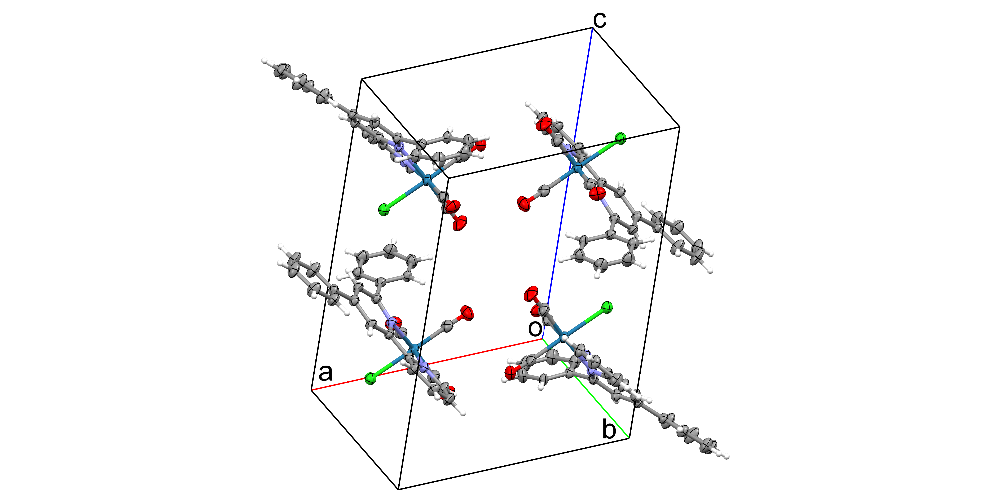 | 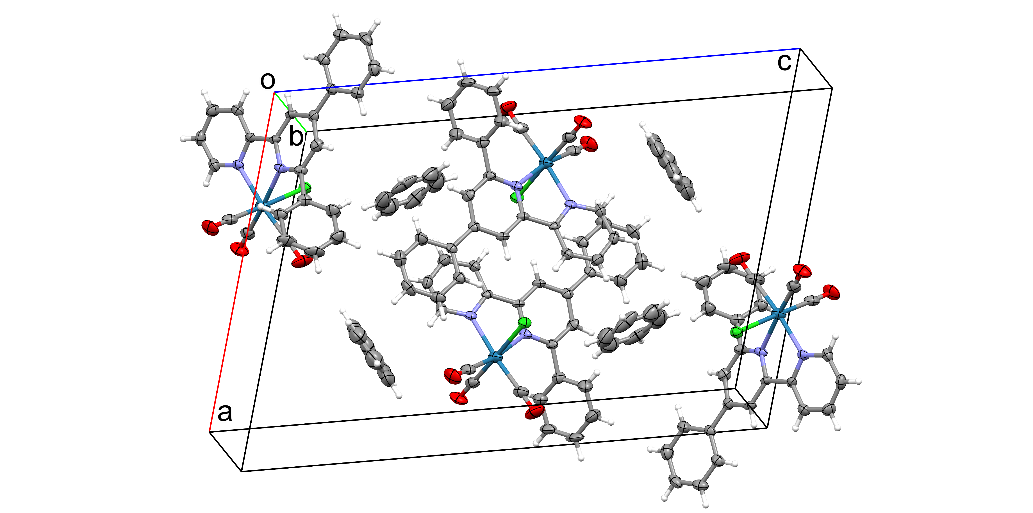 |

**FIGURE S1|** (a) asymmetric unit of **2e** (structure A), (b) asymmetric unit of **2e** (structure B), (c) unit cell of **2e** (structure A) and (d) unit cell of **2e** (structure B).

**TABLE S1**| Crystal data for structure A and B of complex **2e**.

|  | structure A | structure B |
| --- | --- | --- |
| Chemical formula | C_25_H_16_ClN_2_O_3_Re | C_31_H_22_ClN_2_O_3_Re |
| *M*_r_ | 614.06 | 692.17 |
| Crystal system, space group | Monoclinic, *P*2_1_/*c* | Monoclinic, *P*2/*n* |
| Temperature (K) | 293 | 293 |
| *a*, *b*, *c* (Å) | 11.6691 (3), 11.6098 (3), 16.0455 (4) | 15.3042 (3), 7.7657 (1), 23.2383 (5) |
| β (°) | 102.600 (2) | 103.231 (2) |
| *V* (Å^3^) | 2121.43 (9) | 2688.51 (9) |
| *Z* | 4 | 4 |
| Radiation type | Mo *K*α | Mo *K*α |
| µ (mm^−1^) | 5.89 | 4.66 |
| Crystal size (mm) | 0.35 × 0.15 × 0.11 | 0.84 × 0.15 × 0.10 |
| *T*_min_, *T*_max_ | 0.705, 1.000 | 0.437, 0.628 |
| No. of measured, independent and  observed [*I* > 2σ(*I*)] reflections | 12425, 5063, 4444 | 11348, 4720, 3508 |
| *R*_int_ | 0.026 | 0.036 |
| *R*[*F*^2^ > 2σ(*F*^2^)], *wR*(*F*^2^), *S* | 0.023, 0.059, 1.03 | 0.026, 0.066, 1.04 |
| No. of reflections | 5063 | 3508 |
| No. of parameters | 289 | 344 |
| No. of restraints | 0 | 1 |
| Δρ_max_, Δρ_min_ (e Å^−3^) | 0.74, −0.85 | 0.42, −0.37 |

**TABLE S2**| Bond distances (Å) and angles (degree) for **2e** (structure A).

| Atom1 | Atom2 | Length (Å) | Atom1 | Atom2 | Atom3 | Angle (°) |
| --- | --- | --- | --- | --- | --- | --- |
| Re1 | Cl1 | 2.483(1) | Cl1 | Re1 | N2 | 83.03(6) |
| Re1 | N2 | 2.220(2) | Cl1 | Re1 | N1 | 84.14(6) |
| Re1 | N1 | 2.160(2) | Cl1 | Re1 | C1 | 179.0(1) |
| Re1 | C1 | 1.906(4) | Cl1 | Re1 | C2 | 91.3(1) |
| Re1 | C2 | 1.936(5) | Cl1 | Re1 | C3 | 93.8(1) |
| Re1 | C3 | 1.890(3) | N2 | Re1 | N1 | 74.33(9) |
| N2 | C9 | 1.356(4) | N2 | Re1 | C1 | 96.0(1) |
| N2 | C13 | 1.355(4) | N2 | Re1 | C2 | 101.9(1) |
| N1 | C8 | 1.354(3) | N2 | Re1 | C3 | 169.9(1) |
| N1 | C4 | 1.349(4) | N1 | Re1 | C1 | 95.8(1) |
| O2 | C2 | 1.138(5) | N1 | Re1 | C2 | 174.4(1) |
| O1 | C1 | 1.138(5) | N1 | Re1 | C3 | 95.9(1) |
| O3 | C3 | 1.154(4) | C1 | Re1 | C2 | 88.7(2) |
| C8 | C9 | 1.479(4) | C1 | Re1 | C3 | 87.2(1) |
| C8 | C7 | 1.374(5) | C2 | Re1 | C3 | 87.7(2) |
| C9 | C10 | 1.382(3) | Re1 | N2 | C9 | 114.4(2) |
|  | | | Re1 | N2 | C13 | 127.7(2) |
|  |  |  | C9 | N2 | C13 | 117.0(2) |
|  |  |  | Re1 | N1 | C8 | 117.5(2) |
|  |  |  | Re1 | N1 | C4 | 124.8(2) |
|  |  |  | C8 | N1 | C4 | 117.7(3) |
|  |  |  | Re1 | C1 | O1 | 176.1(3) |
|  |  |  | Re1 | C2 | O2 | 179.4(3) |
|  |  |  | N1 | C8 | C9 | 115.2(2) |
|  |  |  | N1 | C8 | C7 | 121.5(3) |
|  |  |  | C9 | C8 | C7 | 123.1(3) |
|  |  |  | N2 | C9 | C8 | 115.5(2) |
|  |  |  | N2 | C9 | C10 | 122.8(3) |
|  |  |  | C8 | C9 | C10 | 121.6(3) |
|  |  |  | Re1 | C3 | O3 | 176.7(3) |

**TABLE S3**| Bond distances (Å) and angles (degree) for **2e** (structure B).

| Atom1 | Atom2 | Length | Atom1 | Atom2 | Atom3 | Angle |
| --- | --- | --- | --- | --- | --- | --- |
| Re1 | Cl1 | 2.486(2) | Cl1 | Re1 | N1 | 85.3(1) |
| Re1 | N1 | 2.177(4) | Cl1 | Re1 | N2 | 81.6(1) |
| Re1 | N2 | 2.233(5) | Cl1 | Re1 | C2 | 90.8(2) |
| Re1 | C2 | 1.952(7) | Cl1 | Re1 | C3 | 91.1(2) |
| Re1 | C3 | 1.933(7) | Cl1 | Re1 | C1 | 178.5(2) |
| Re1 | C1 | 1.919(7) | N1 | Re1 | N2 | 74.6(2) |
| N1 | C8 | 1.352(8) | N1 | Re1 | C2 | 174.9(3) |
| N1 | C4 | 1.338(8) | N1 | Re1 | C3 | 97.2(2) |
| N2 | C13 | 1.338(8) | N1 | Re1 | C1 | 93.2(2) |
| N2 | C9 | 1.364(7) | N2 | Re1 | C2 | 101.6(3) |
| O2 | C2 | 1.116(9) | N2 | Re1 | C3 | 169.3(2) |
|  | | | N2 | Re1 | C1 | 98.0(2) |
|  |  |  | C2 | Re1 | C3 | 86.1(3) |
|  |  |  | C2 | Re1 | C1 | 90.7(3) |
|  |  |  | C3 | Re1 | C1 | 89.1(3) |
|  |  |  | Re1 | N1 | C8 | 116.3(4) |
|  |  |  | Re1 | N1 | C4 | 124.7(4) |
|  |  |  | C8 | N1 | C4 | 118.8(5) |
|  |  |  | Re1 | N2 | C13 | 127.5(4) |
|  |  |  | Re1 | N2 | C9 | 113.2(4) |
|  |  |  | C13 | N2 | C9 | 117.7(5) |
|  |  |  | Re1 | C2 | O2 | 177.2(7) |
|  |  |  | N2 | C13 | C12 | 121.9(6) |
|  |  |  | N2 | C13 | C14 | 119.7(6) |

| (a) | (b) | (c) |
| --- | --- | --- |
| 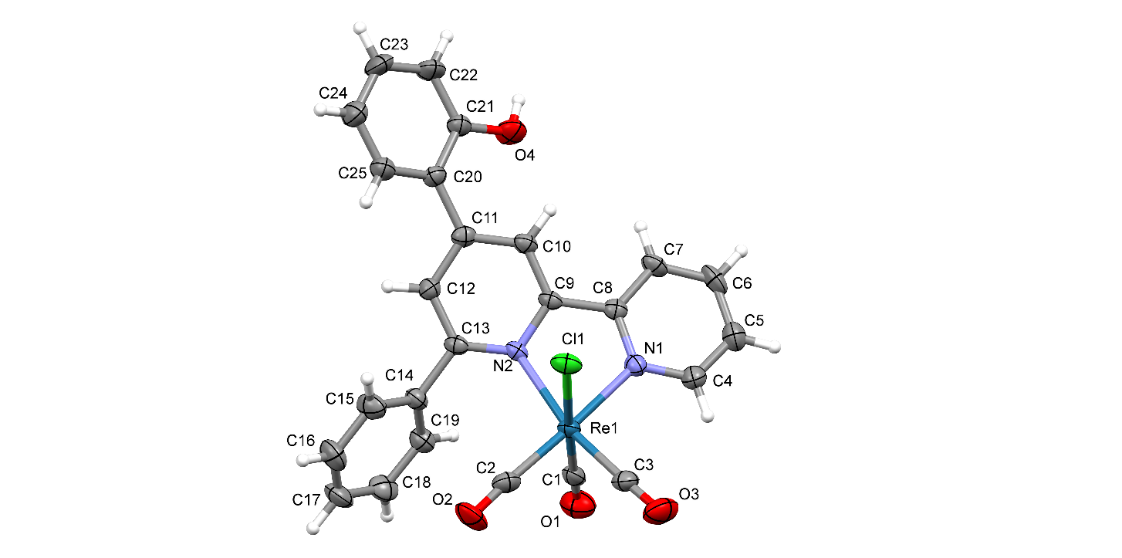 | 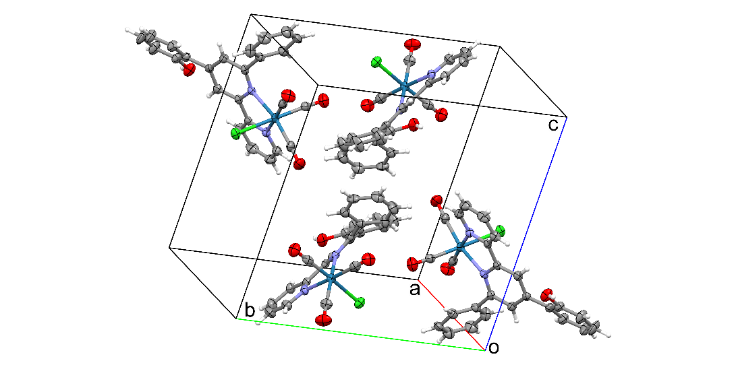 | ~~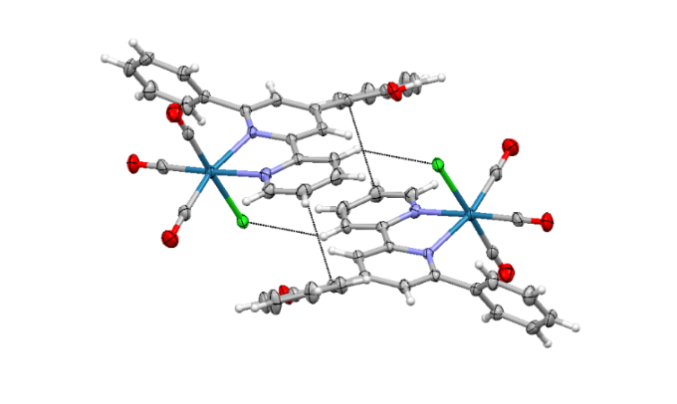~~ |

**FIGURE S2|** (a) asymmetric unit, (b) unit cell and (c) C–H···Cl and π···π stacking contacts in the crystal structure of **2f**.


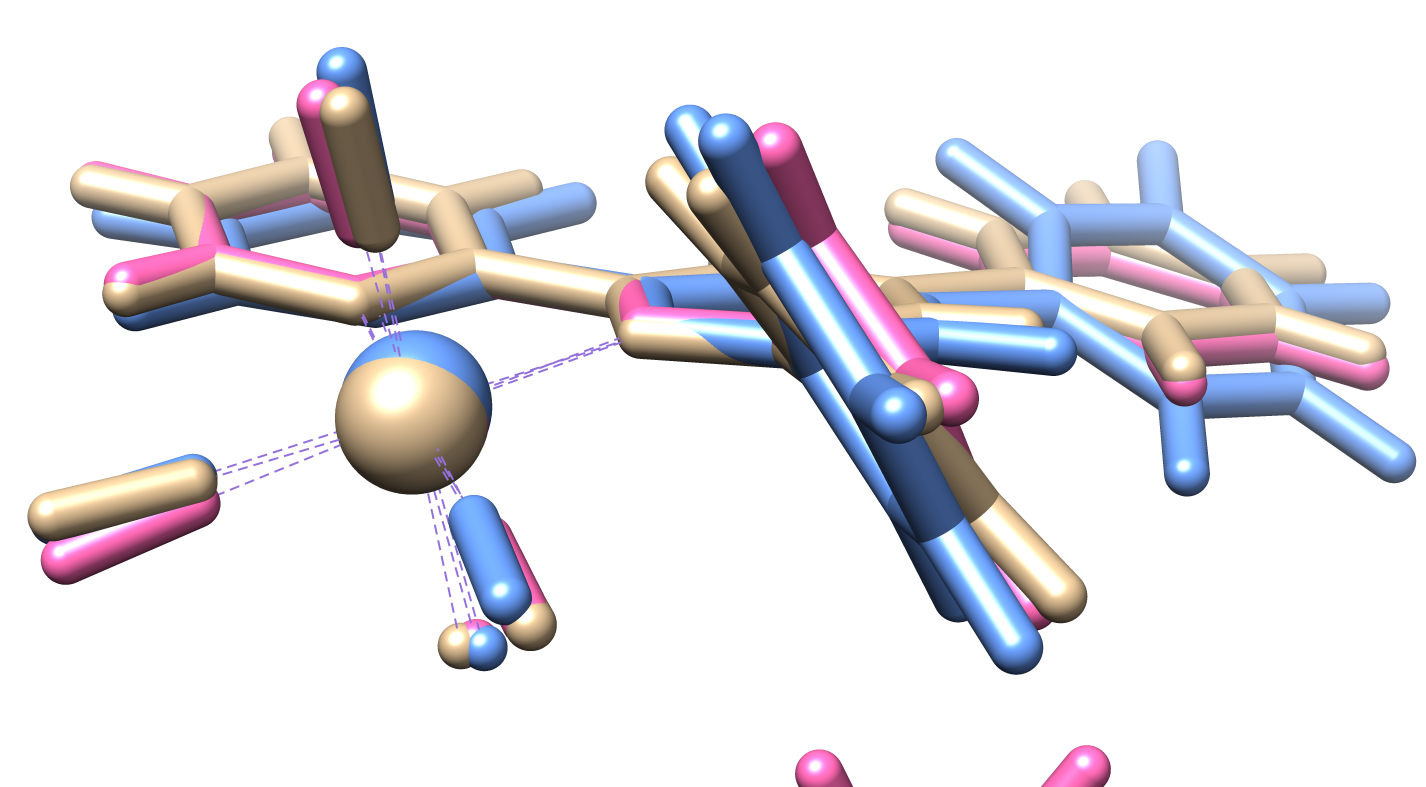


**Figure S3|** Comparison of DFT optimized (light blue) and X-ray structure A (brown) and B (pink) of **2e**.

**TABLE S4**| data for **2f**.

| Chemical formula | C_25_H_16_ClN_2_O_4_Re |
| --- | --- |
| *M*_r_ | 630.06 |
| Crystal system, space group | Monoclinic, *P*2_1_/*n* |
| Temperature (K) | 293 |
| *a*, *b*, *c* (Å), β (°) | 9.6474 (6), 15.8756 (10), 14.8432 (8), 93.292 (5) |
| *V* (Å^3^) | 2269.6 (2) |
| *Z* | 4 |
| Radiation type | Mo *K*α |
| µ (mm^−1^) | 5.51 |
| Crystal size (mm) | 0.53 × 0.37 × 0.22 |
|  | |
| No. of measured, independent and observed reflections | 10670, 3786, 3073 |
| *R*_int_ | 0.047 |
| *R*[*F*^2^ > 2σ(*F*^2^)], *wR*(*F*^2^), *S* | 0.031, 0.073, 1.02 |
| No. of reflections and parameters | 3786, 299 |
| Δρ_max_, Δρ_min_ (e Å^−3^) | 0.96, −1.01 |

**TABLE S5**| Bond distances (Å) and angles (degree) for **2f**.

| Atom1 | Atom2 | Length (A°) | Atom1 | Atom2 | Atom3 | Angle (degree) |
| --- | --- | --- | --- | --- | --- | --- |
| Re1 | Cl1 | 2.490(2) | Cl1 | Re1 | N2 | 80.2(1) |
| Re1 | N2 | 2.214(4) | Cl1 | Re1 | N1 | 85.1(1) |
| Re1 | N1 | 2.164(5) | Cl1 | Re1 | C1 | 177.1(2) |
| Re1 | C1 | 1.921(6) | Cl1 | Re1 | C2 | 94.9(2) |
| Re1 | C2 | 1.935(6) | Cl1 | Re1 | C3 | 91.0(2) |
| Re1 | C3 | 1.898(6) | N2 | Re1 | N1 | 74.9(2) |
| O1 | C1 | 1.106(8) | N2 | Re1 | C1 | 99.0(2) |
| O2 | C2 | 1.137(8) | N2 | Re1 | C2 | 102.1(2) |
| O3 | C3 | 1.158(8) | N2 | Re1 | C3 | 167.3(2) |
| N1 | C4 | 1.338(8) | N1 | Re1 | C1 | 92.0(2) |
| C7 | C6 | 1.378(9) | N1 | Re1 | C2 | 177.0(2) |
| C20 | C11 | 1.482(7) | N1 | Re1 | C3 | 95.3(2) |
| C20 | C21 | 1.398(8) | C1 | Re1 | C2 | 88.0(3) |
| C20 | C25 | 1.383(8) | C1 | Re1 | C3 | 89.4(3) |
| C11 | C12 | 1.397(8) | C2 | Re1 | C3 | 87.6(3) |
| C11 | C10 | 1.378(7) | Re1 | C1 | O1 | 177.3(5) |
| C13 | C12 | 1.382(8) | Re1 | C2 | O2 | 176.7(5) |
| O4 | C21 | 1.357(8) | Re1 | C3 | O3 | 177.5(6) |
|  | | | Re1 | N1 | C8 | 116.5(3) |
|  |  |  | Re1 | N1 | C4 | 124.9(4) |

**TABLE S6**| Contact table for **2f**.

| Atom1 | Atom2 | Length | Length-VdW | Symm. op. 1 | Symm. op. 2 |
| --- | --- | --- | --- | --- | --- |
| O4 | Cl1 | 3.117 | -0.153 | x,y,z | -1+x,y,z |
| O4 | C2 | 3.142 | -0.078 | x,y,z | -1+x,y,z |
| H23 | N2 | 2.750 | -0.000 | x,y,z | -1/2-x,-1/2+y,1/2-z |
| Cl1 | H7 | 2.864 | -0.086 | x,y,z | -x,-y,1-z |
| C20 | C5 | 3.395 | -0.005 | x,y,z | -x,-y,1-z |
| H6 | O1 | 2.515 | -0.205 | x,y,z | -1/2+x,1/2-y,1/2+z |

The crystal packing of the different structures shows some interesting trend that can be analyzed with the use of Hirshfeld Surface (Hirshfeld, 1977) calculated using the program CrystalMaker (McKinnon et al., 2004, 2007). This is a powerful tool to show and analyze the intermolecular interaction, and by checking its properties and the fingerprint generated for the different interaction components it is possible to study the packing forces in a more physically correct way than only checking the interatomic distances. The Hirshfeld surface of a molecule or a fragment is the surface that enclose the volume in the crystal space in which the electron density of that fragment or molecule is the majority of the electron density. On this surface, some properties can be shown: for our analysis it will be considered the normalized distance d_norm_, that is the distance between a point on the surface and the nearest atom weighted for the Van der Waals radius of this atom, very useful to check the presence of hydrogen bonds, and the shape index, a peculiar alternative definition of the second derivatives (curvatures) of a surface, very sensitive to low differences in electron density distributions and very useful to check the presence and location of π···π interactions (McKinnon et al., 2004, 2007). On the other hand, Fingerprints have been used and their decomposition: this graphical instrument is created by projecting on a plane the value of the distance of each point of the surface from the nearest internal (d_i_) and external (d_e_) (McKinnon et al., 2004, 2007). This fingerprint can be decomposed in its component related to different contacts, and the form of this fingerprints is very diagnostic of the presence and strength of the different interactions. By analyzing the two structures obtained for **2e** with these instruments, the main packing forces are weak hydrogen bonds and π···π interactions. Red spots in the d_norm_ surfaces near the Cl and CO fragments in the directions of C–H bonds are the signal of presence of weak C–H···Cl and C–H···O interactions in both the structures (Figure S4c for A and Figure S6a,b for B). However, in structure B a strong component of C–H···π interaction can be seen due to the presence of benzene molecules perpendicular to the ligand structure (Figure S6a). All these interactions can be seen also in the decomposition of the fingerprints in the two wings and tails symmetric respect to the diagonal (Figure S5c-e for structure A and Figure S7c-e for structure B). The π···π interactions are present along the structure of the ligand, especially the phenyl substitution in the 4 position, and can be seen in the presence of blue and red triangles in the shape index surface especially for B (Figure S6c-d) but are also significant in Figure S4b along the two ligands. This interaction is indicated in the fingerprint by a central node of C···C contacts in Figure S5b for A and Figure S7b for B. It is interesting to consider, however, the percentage of the interactions in the fingerprint, that is a measure of the importance of these in the packing of the crystal. The π···π stacking account for the 8% in B, while is 5% in A of the overall Hirshfeld surface, while all the hydrogen bond components increase passing from B to A. This fact, and the derived disposition of the molecules in the solids, can be hypothetically correlated to the polarity of the crystal growth solvents, that makes favorable dispersion forces in presence of benzene, while hydrogen bonds became dominants in presence of polar solvents like acetones. In the case of **2f**, the crystal packing is dominated by shorter directional C–H···O and C–H···Cl contacts visible in the fingerprint (Figure S9d,e) in directions parallel to the chain, while π···π stacking seems to be less present but still fundamental due to coupling of inverted dipole of molecules (Figure S2c).

| (a)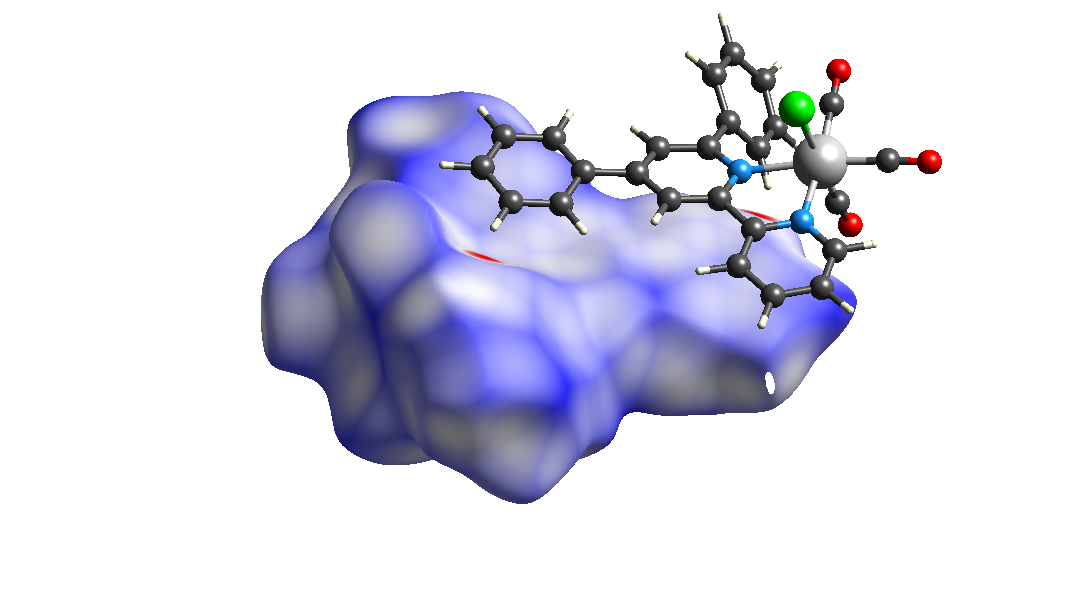 | (b)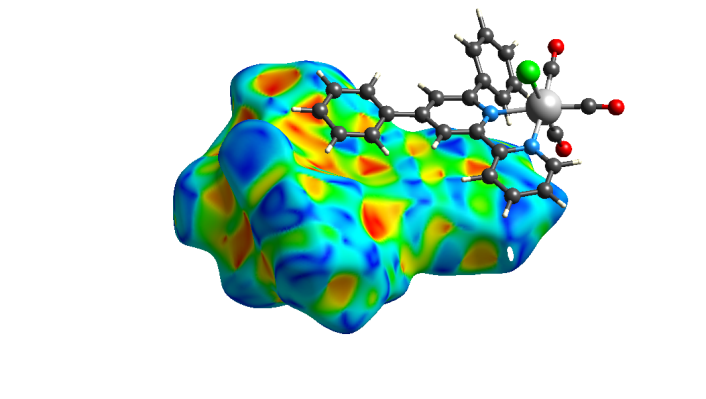 | (c)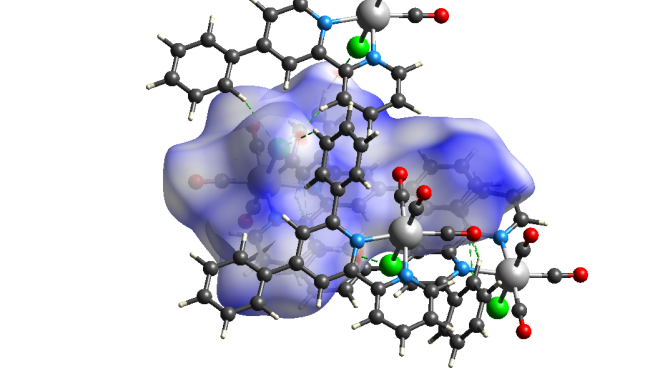 |
| --- | --- | --- |

**FIGURE S4|** (a-c) representation of the d_norm_ property and (b) representation of the shape index property on the Hirshfeld surface of the molecule in crystal **2e** (structure A).

| (a)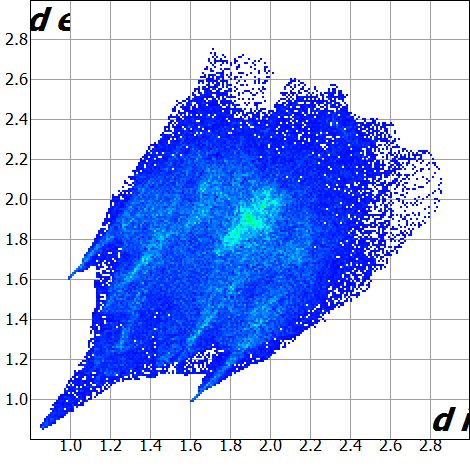 | (b)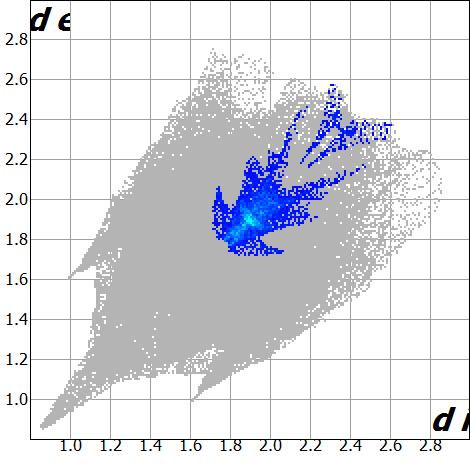 | | (c)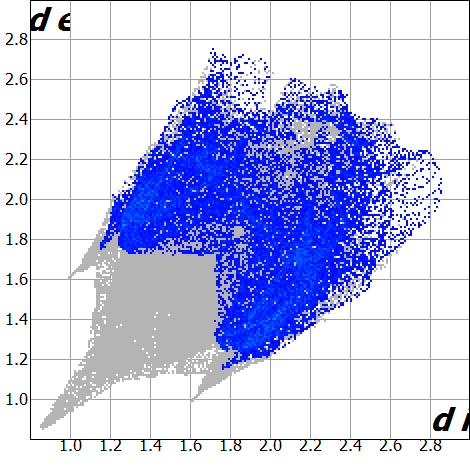 |
| --- | --- | --- | --- |
| (d)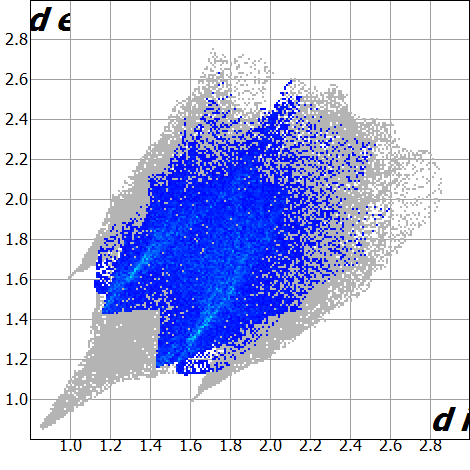 | | (e)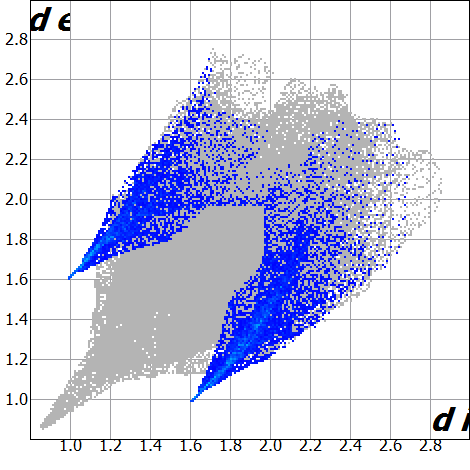 | |

**FIGURE S5|** Fingerprints generated by the Hirshfeld surface of crystal **2e** (structure A). (a) and decomposition of its components for the main interactions present in the structure: (b) π···π interaction, (c) C–H···π interaction, (d) C–H···O interaction and (e) C–H···Cl interaction.

| (a)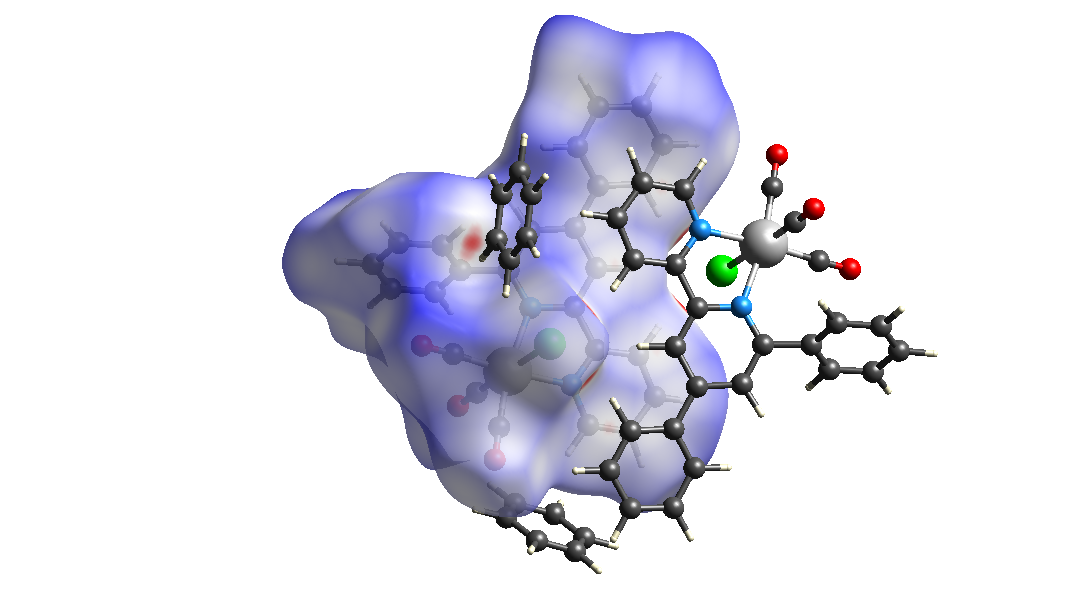 | (b)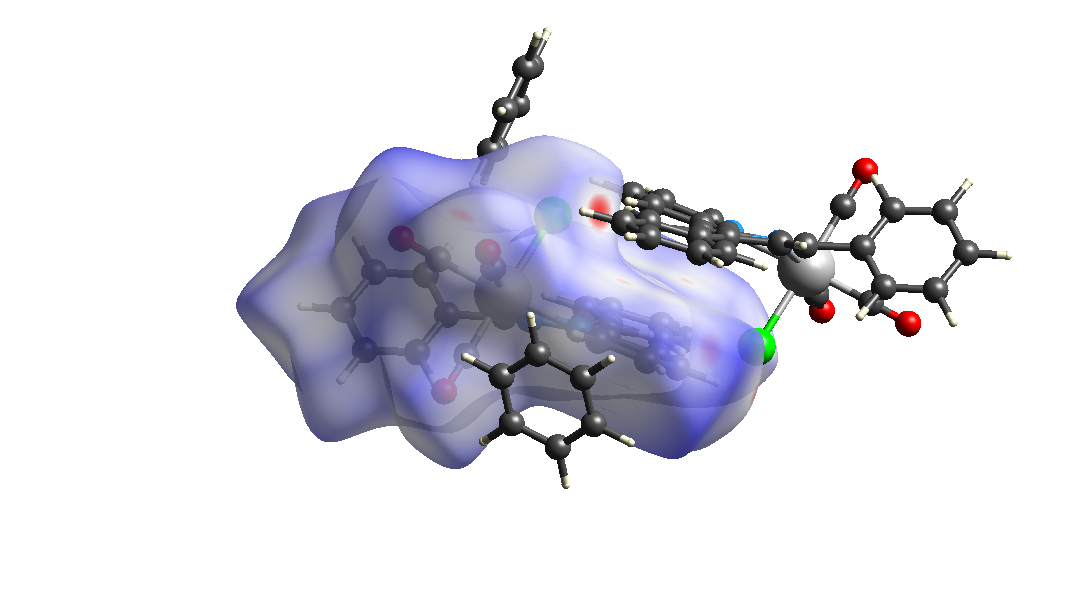 |
| --- | --- |
| (c)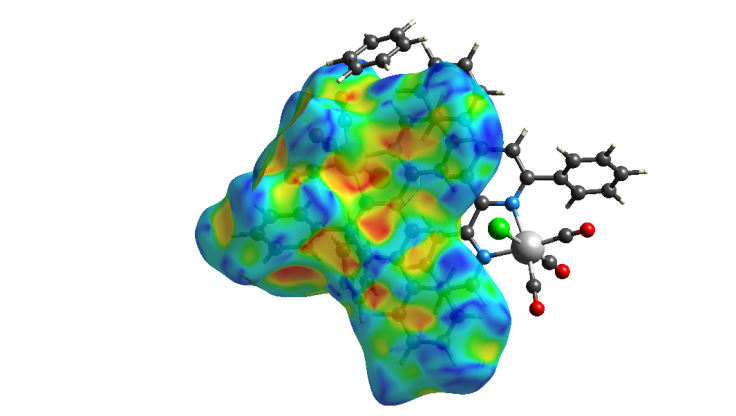 | (d)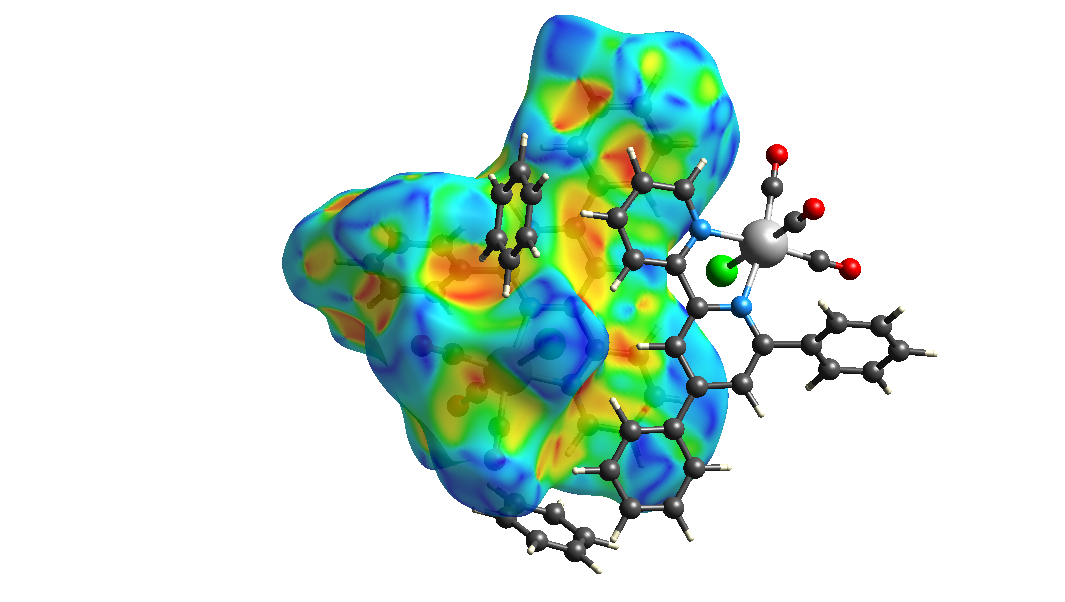 |

**FIGURE S6|** (a-b) representation of the d_norm_ property and (b-c) representation of the shape index property on the Hirshfeld surface of the molecule in crystal **2e** (structure B).

| (a)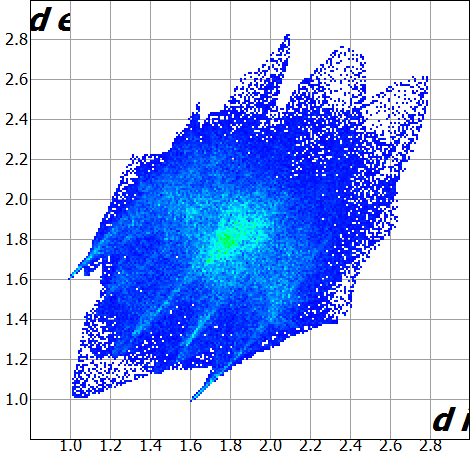 | (b)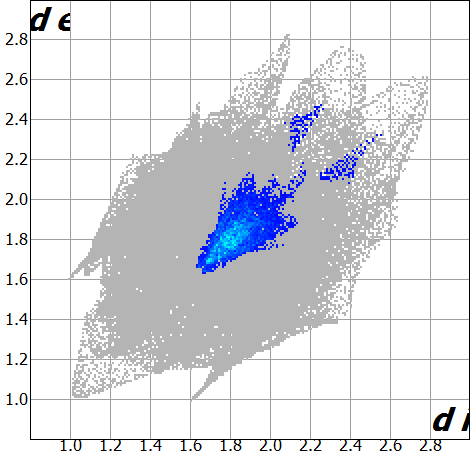 | | (c)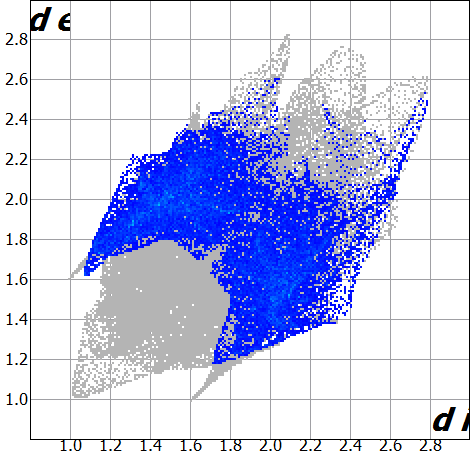 |
| --- | --- | --- | --- |
| (d)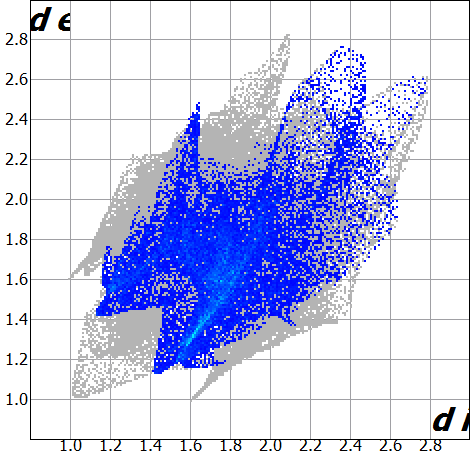 | | (e)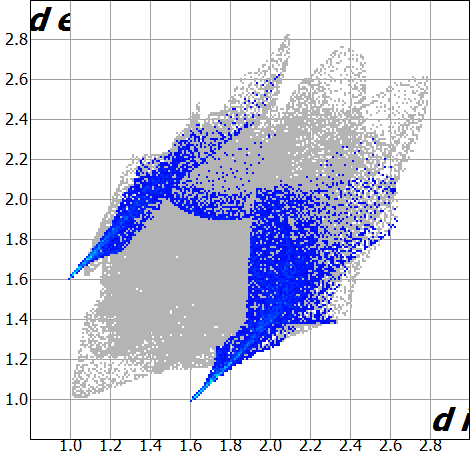 | |

**FIGURE S7|** Fingerprints generated by the Hirshfeld surface of crystal **2e** (structure B) (a) and decomposition of its components for the main interactions present in the structure: (b) π···π interaction, (c) C–H···π interaction, (d) C–H···O interaction and (e) C–H···Cl interaction.

| (a)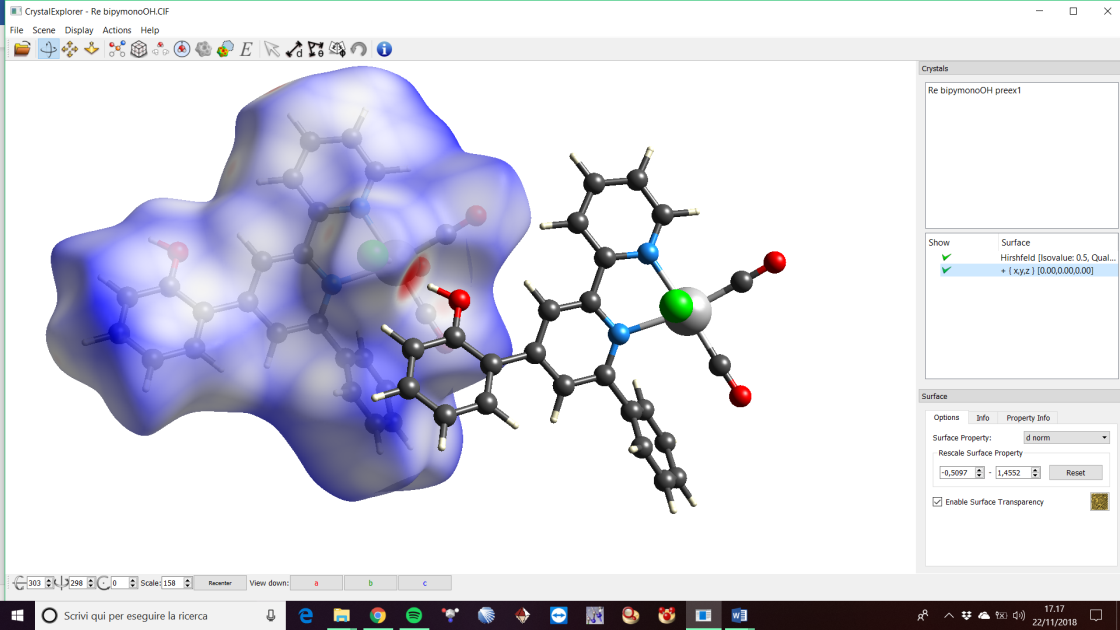 | (b)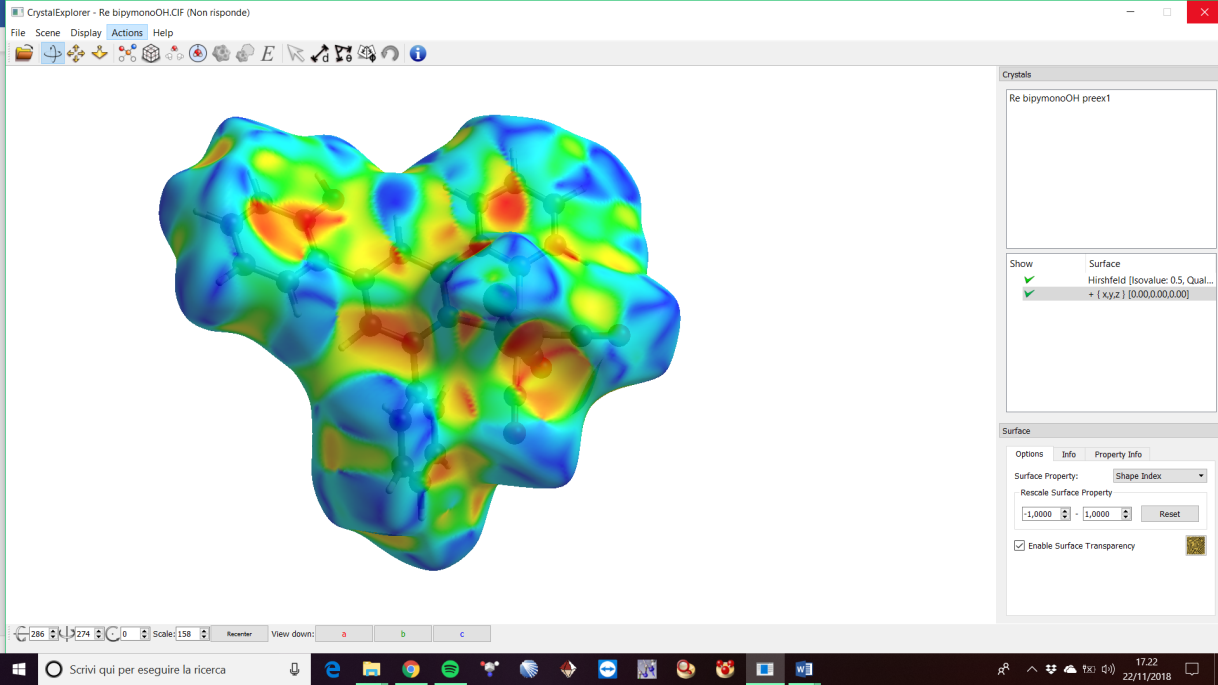 |
| --- | --- |

**FIGURE S8|** (a) representation of the d_norm_ property and (b) representation of the shape index property on the Hirshfeld Surface of the molecule in crystal **2f**.

| (a)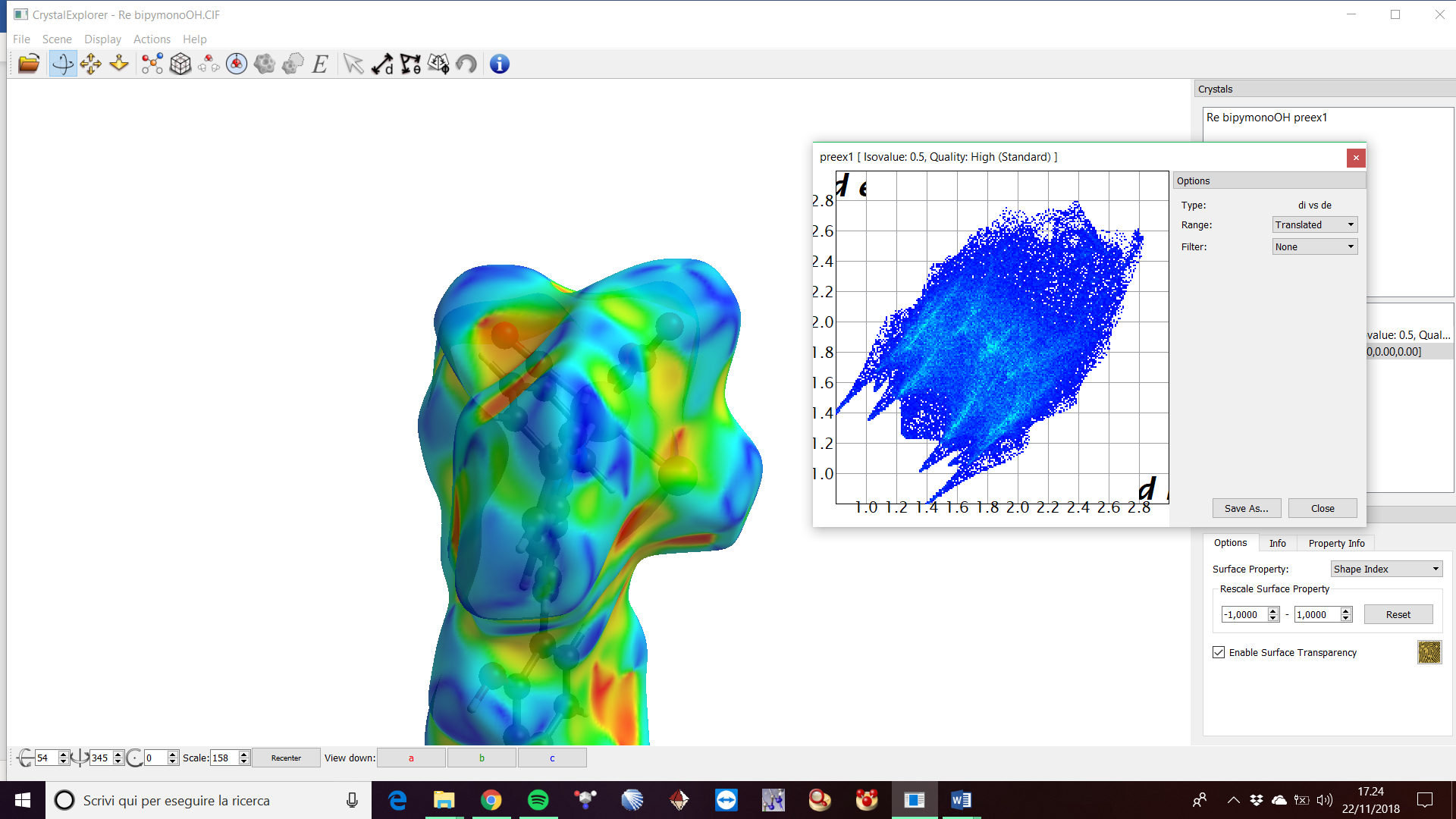 | (b)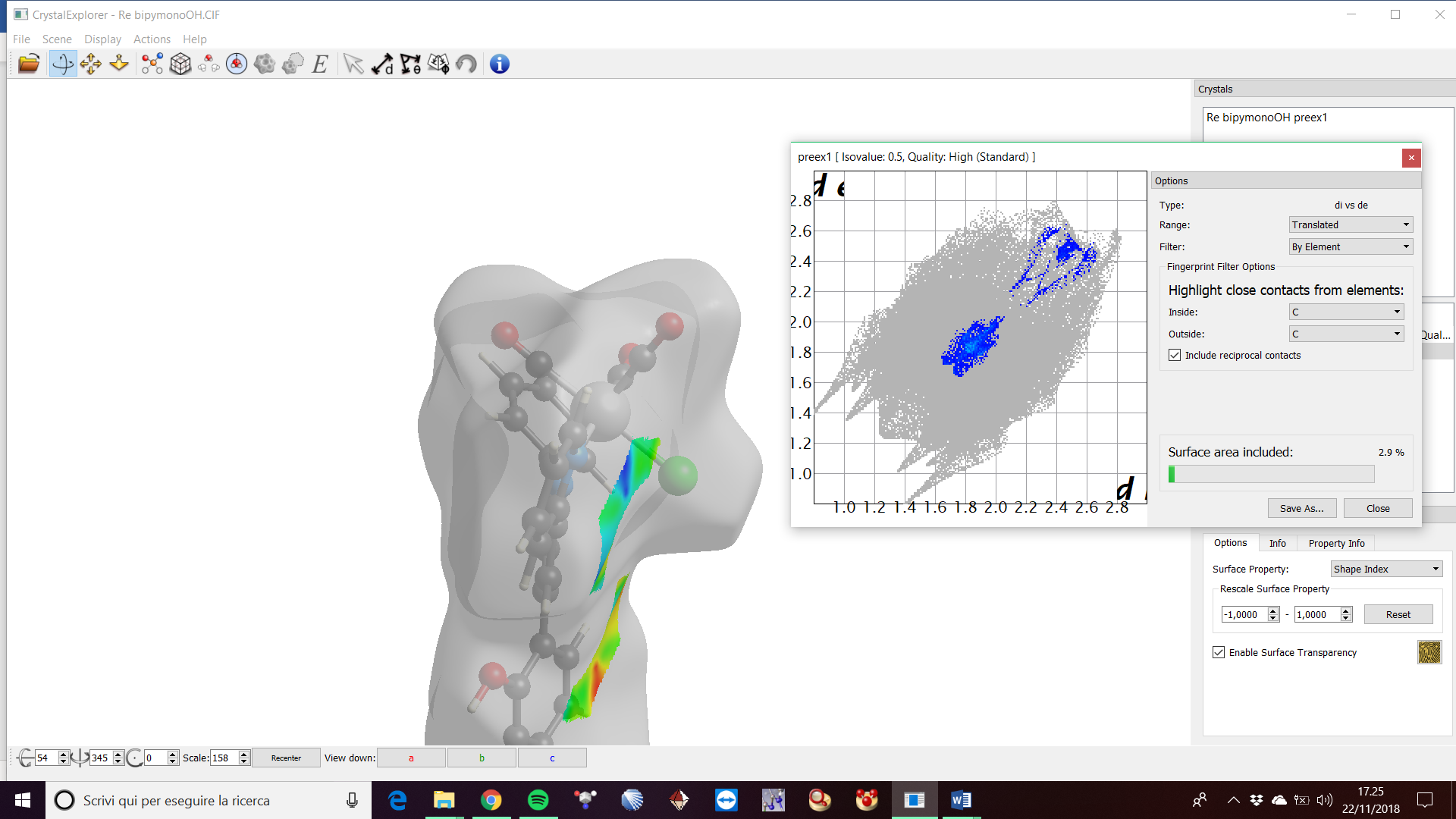 | | (c)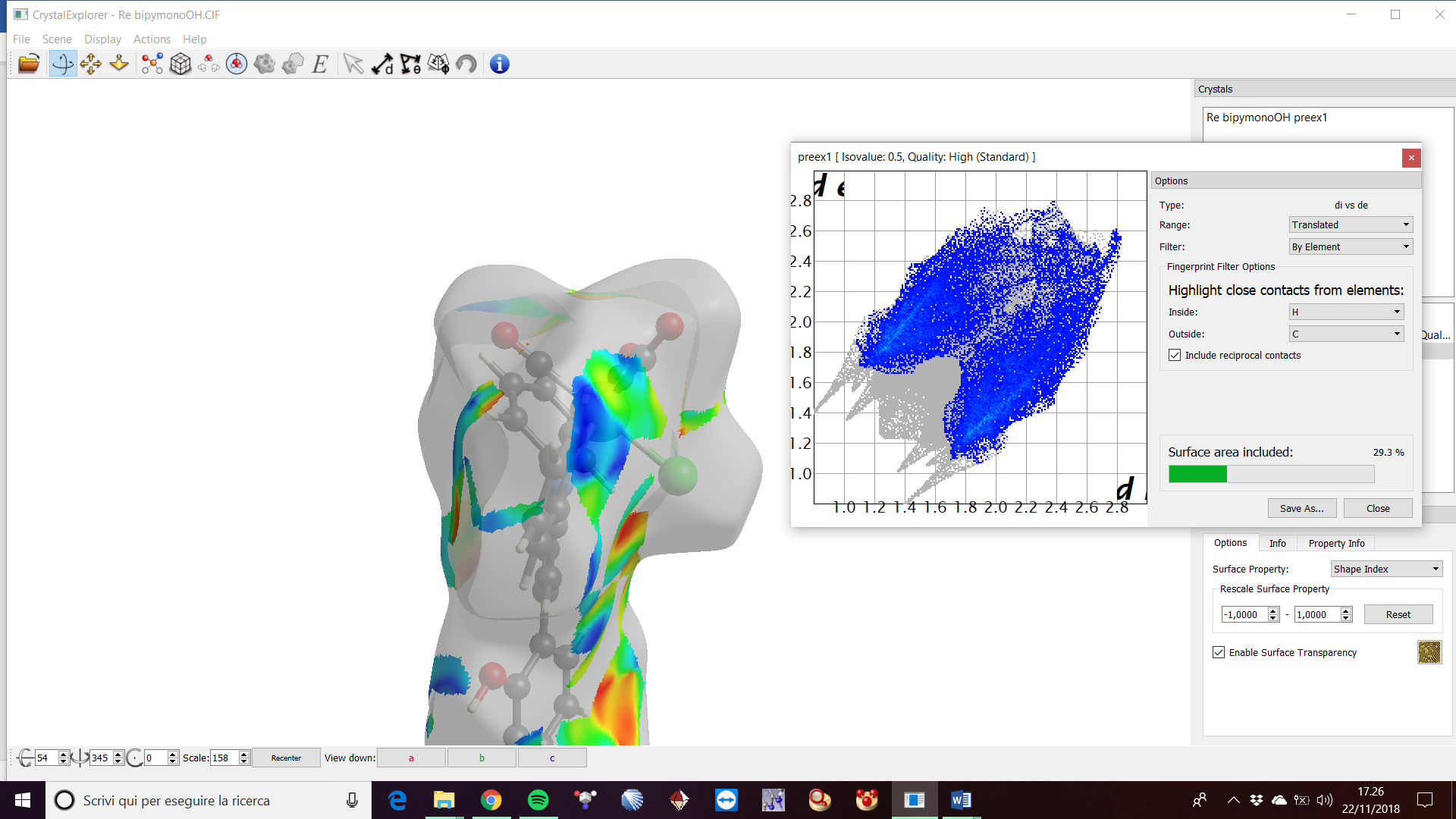 |
| --- | --- | --- | --- |
| (d)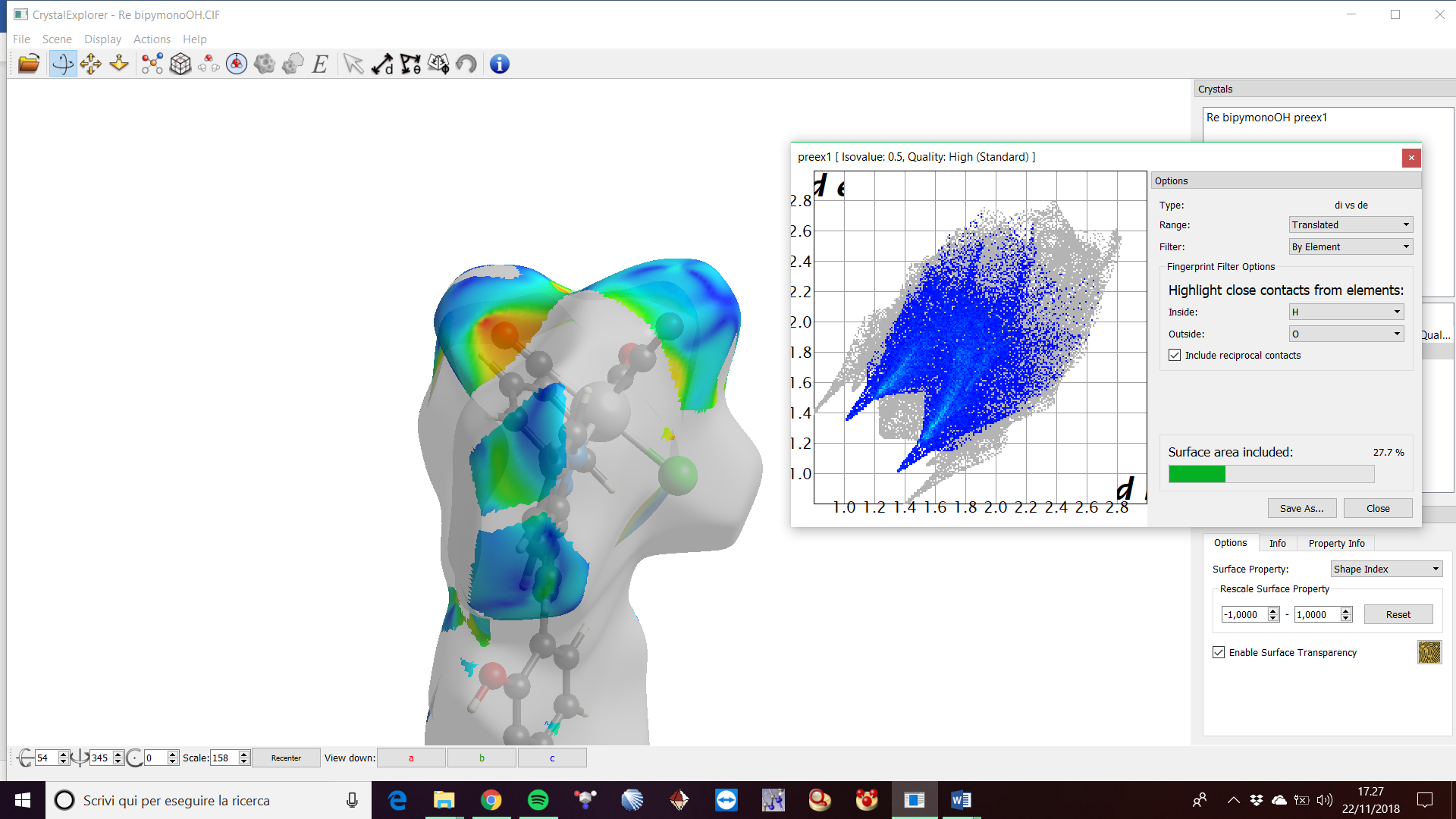 | | (e)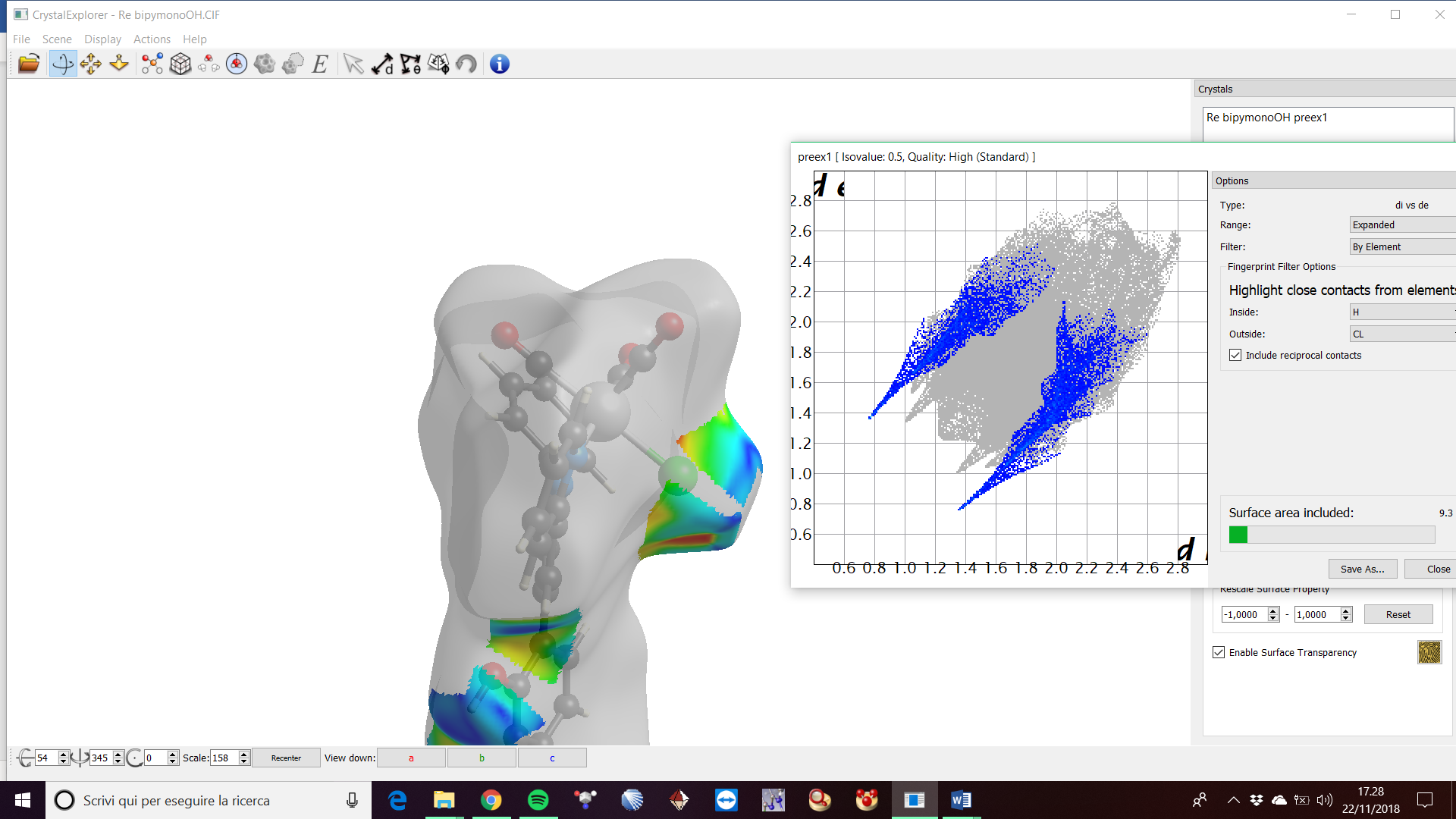 | |

**FIGURE S9|** Fingerprints generated by the Hirshfeld surface of crystal **2f**. (a) and decomposition of its components for the main interactions present in the structure: (b) π···π interaction, (c) C–H···π interaction, (d) C–H···O interaction and (e) C–H···Cl interaction.





**FIGURE S10|** CVs of 1mM solution of **1a** in MeCN/0.1M TBAPF_6_ at GCE, scan rate 200 mVs^-1^ under Ar.





**FIGURE S11|** CVs of 1mM solution of **1a** in MeCN/0.1M TBAPF_6_ at GCE, scan rate 1Vs^-1^ under Ar.


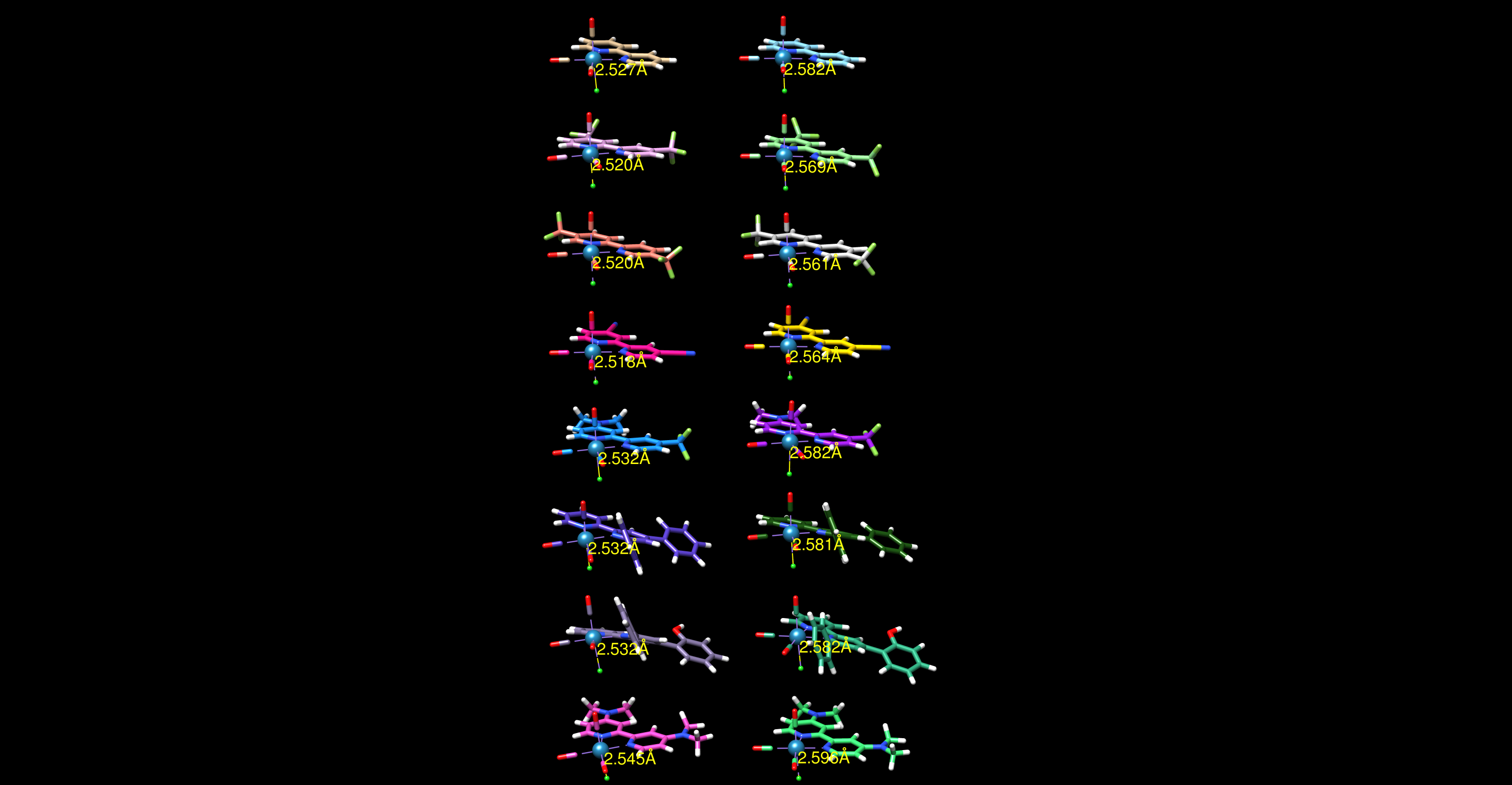


**Figure S12|** DFT optimized structures of the investigated Re complexes in their ground state (left) and reduced (right) form.


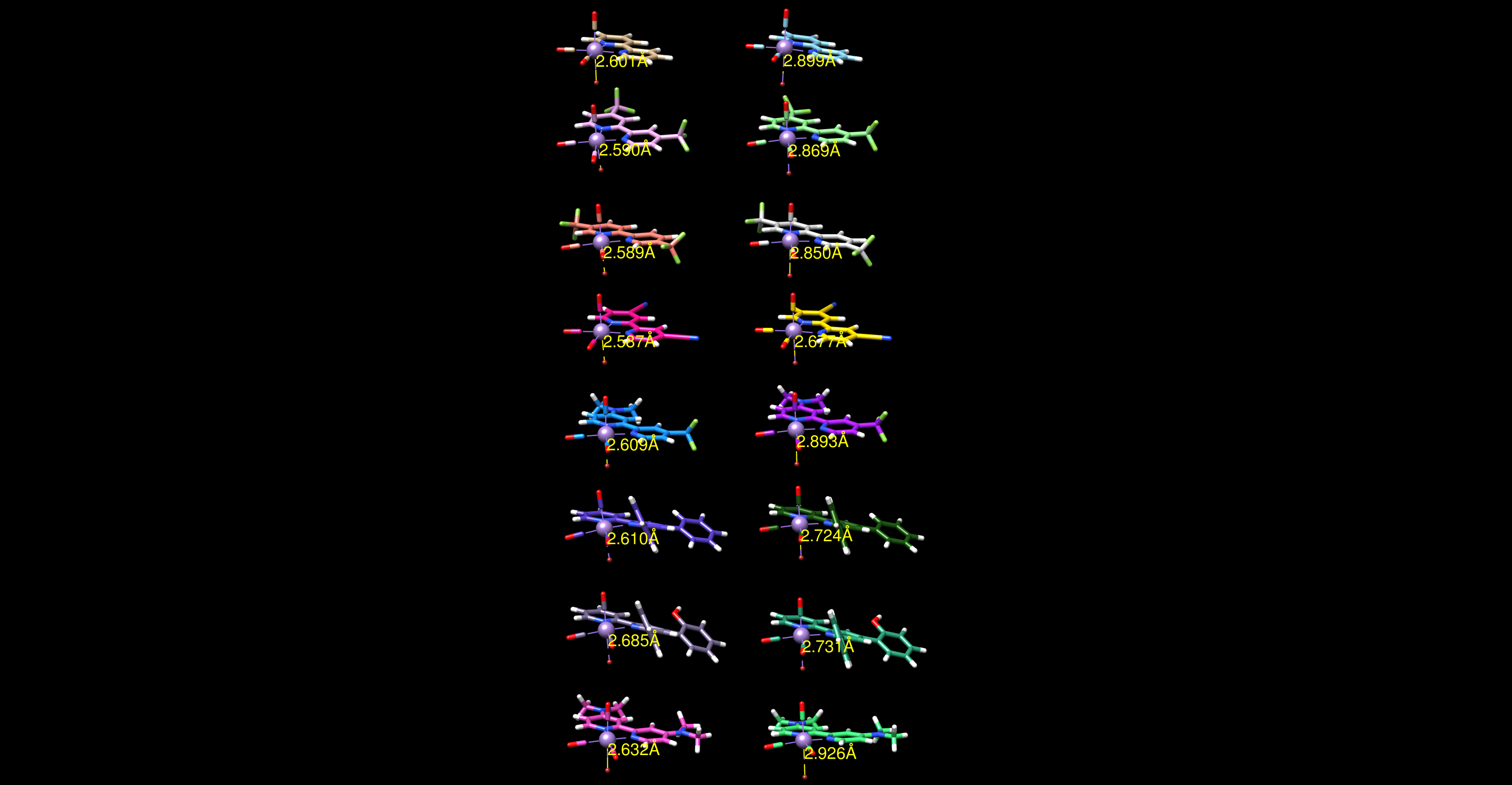


**Figure S13|** DFT optimized structures of the investigated Mn complexes in their ground state (left) and reduced (right) form.


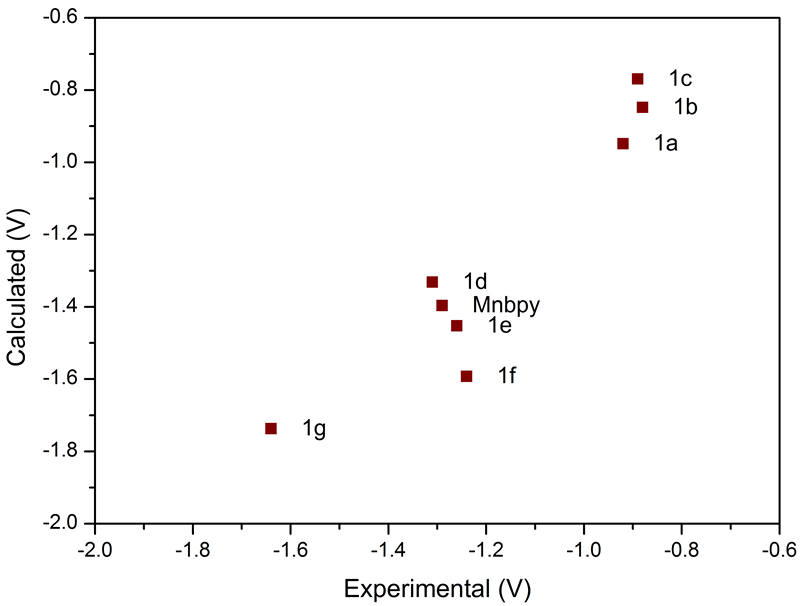


**FIGURE S14|** Plot of the calculated *vs.* experimental standard reduction potentials for **1a-1g**.

| **__** | **__** |
| --- | --- |
|  |  |
|  |  |
|  |  |

**FIGURE S15|** CVs of 0.5 mM solutions of **1a-1g** Mn complexes in MeCN/0.1M TBAPF_6_ at GCE, scan rate 200 mVs^-1^ under Ar, under CO_2_ and for **1d-1g** + 5%v MeOH. CV in black is the electrolyte saturated with CO_2_.

|  |  |
| --- | --- |
|  |  |
|  |  |
| 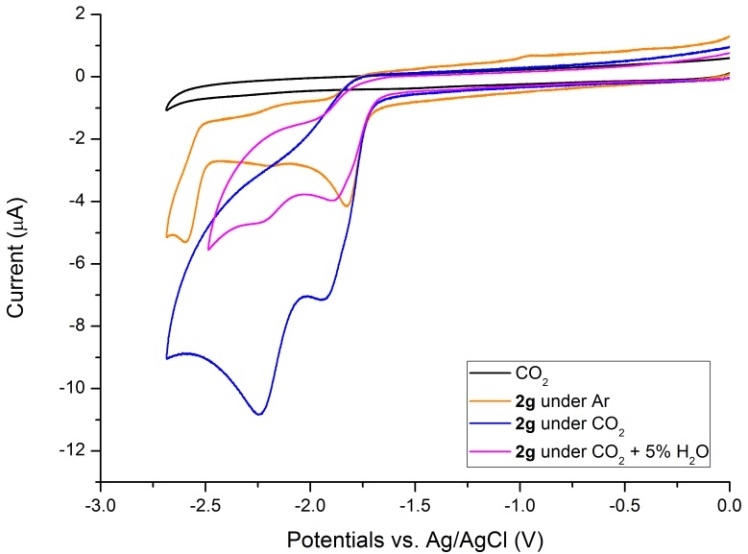 |  |

**FIGURE S16|** CVs of 0.5 mM solutions of **2a-2g** Re complexes in MeCN/0.1M TBAPF_6_ at GCE, scan rate 200 mVs^-1^ under Ar, under CO_2_ and + 5%v H_2_O. CV in black is the electrolyte saturated with CO_2_.

**TABLE S7**| TON and faradic efficiencies (η) upon CPE (applied potential E in V vs. Ag/AgCl) of solutions of manganese and rhenium complexes (0.5 mM) in 0.1 M TBAPF_6_/MeCN in the presence of Brønsted acids (5%v).

| **Complex** | **E [V]** | **T [min]** | **acid [5%]** | **TON_CO_** | **η_CO_ [%]** | **TON_H2_** | **η_H2_ [%]** |
| --- | --- | --- | --- | --- | --- | --- | --- |
| **1d** | -1.5 | 90 | MeOH | 0.5 | - | - | - |
| **1e** | -1.5 | 60 | MeOH | 0.7 | - | - | - |
| **1f** | -1.5 | 300 | MeOH | 1.5 | - | - | - |
| **1g** | -1.95 | 60 | MeOH | 2.6 | 85 | - | - |
| **2d** | -1.8 | 60 | - | 0.5 | - | - | - |
| **2d** | -1.8 | 300 | H_2_O | 2 |  | - | - |
| **2e** | -1.7 | 120 | - | 1 | - | - | - |
| **2e** | -1.7 | 120 | H_2_O | 1.5 | - | - | - |
| **2f** | -1.7 | 360 | H_2_O | 14 | 85 | - | - |
| **2g** | -2 | 300 | H_2_O | 23 | 100 | - | - |

| 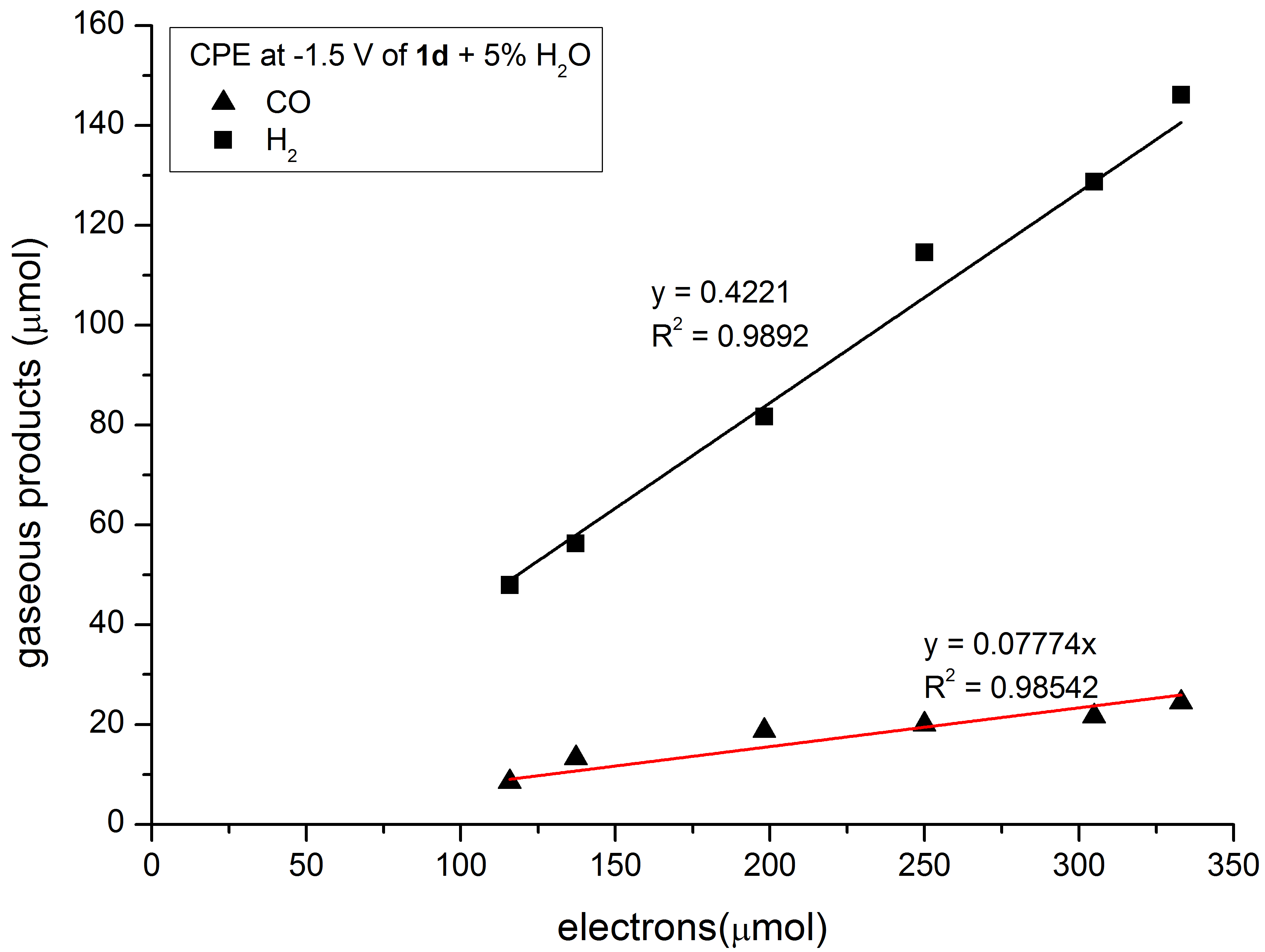 | 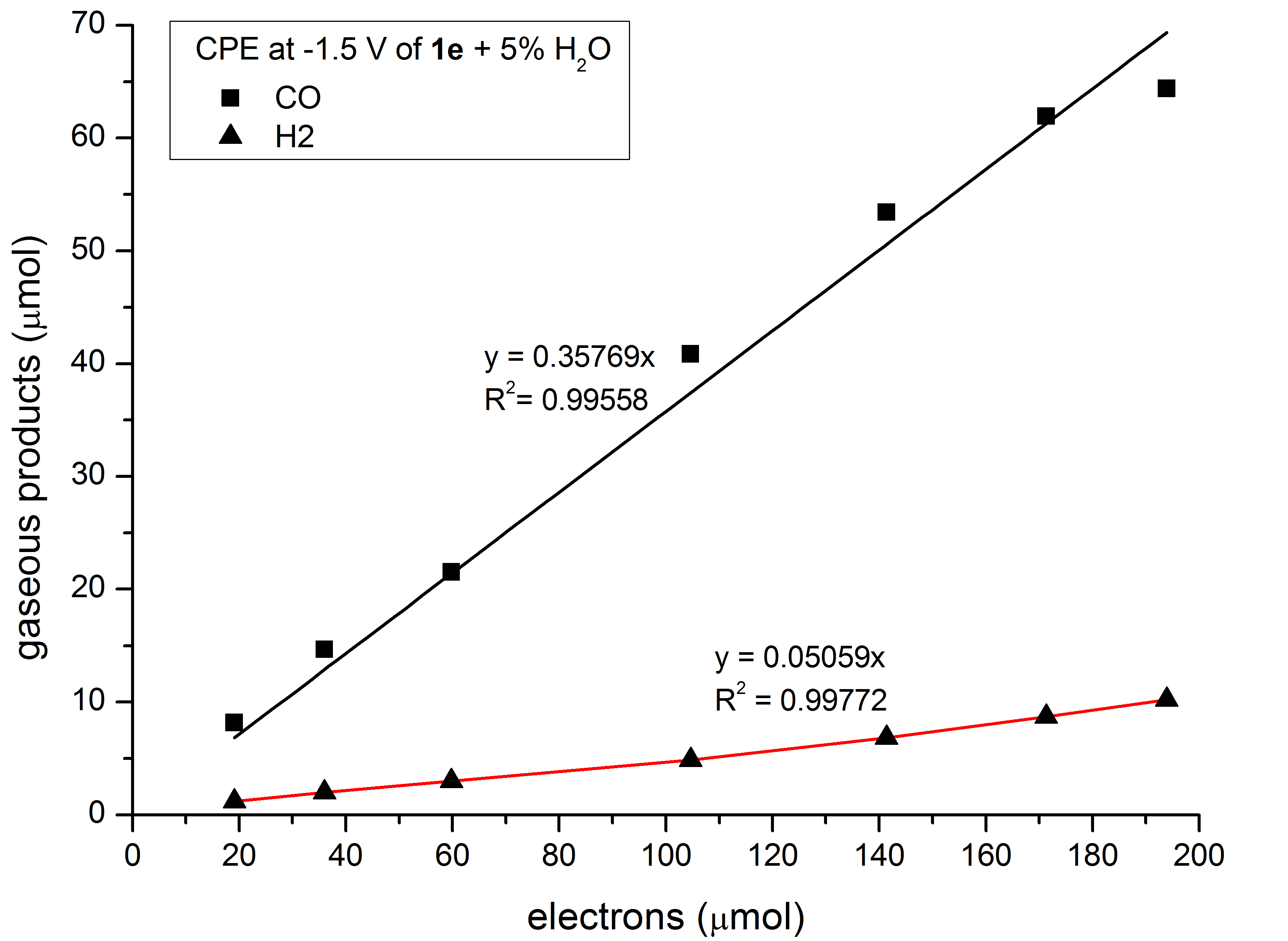 |
| --- | --- |
|  |  |

**FIGURE S17|** Catalytic CO and H_2_ production from CO_2_ as a function of the charge during CPE carried out in MeCN solutions of **1d-1g** (0.5 mM) with 5%v H_2_O.

|  |  |
| --- | --- |
|  | 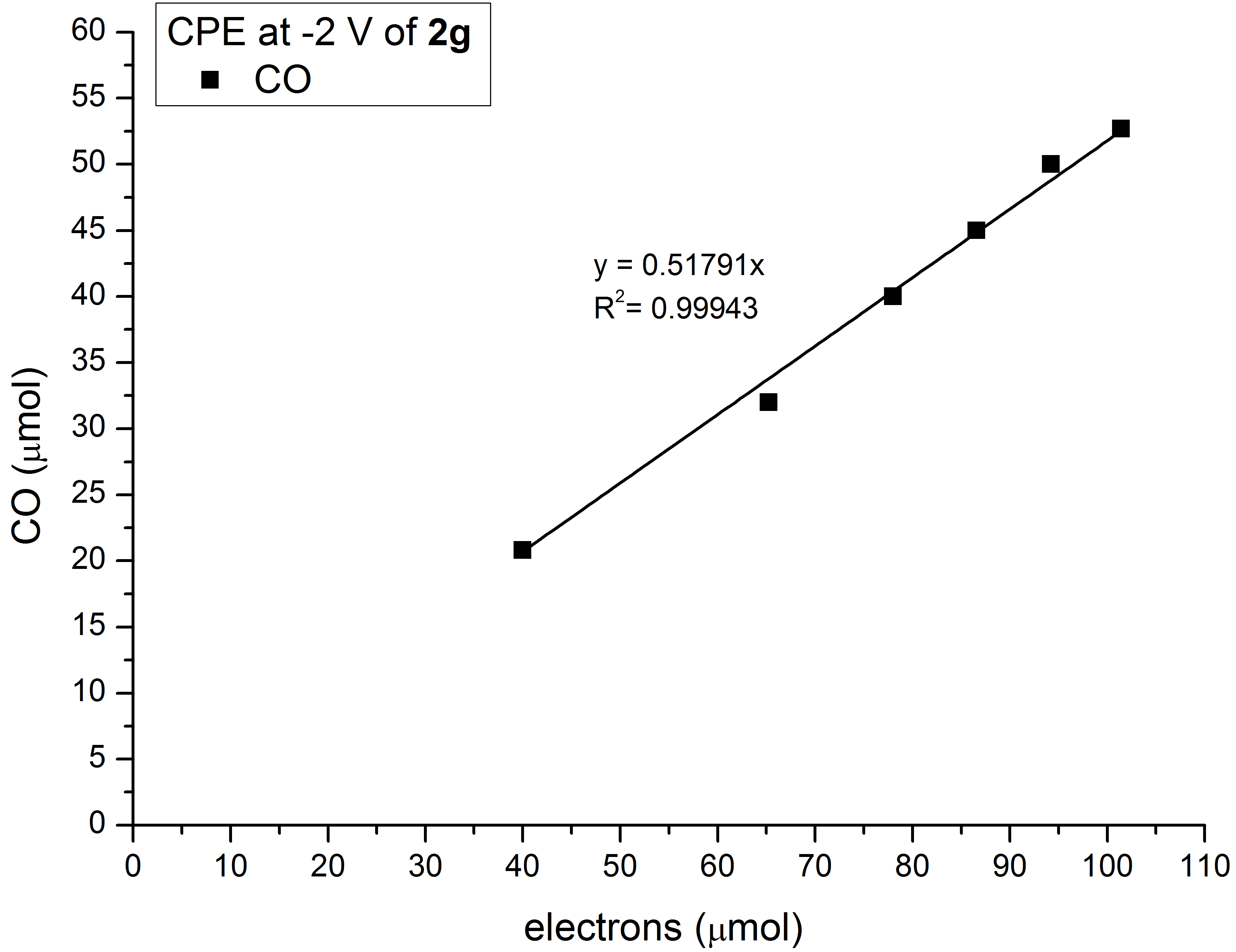 |
|  |  |

**FIGURE S18|** Catalytic CO production from CO_2_ as a function of the charge during CPE carried out in MeCN solutions of **2e-2g** (0.5 mM) both in absence and presence of 5%v MeOH.

**NMR assignments of ligands a-g:**

Ligand **a (**4,4'-bis(trifluoromethyl)-2,2'-bipyridine) ^1^H NMR [600 MHz, CDCl_3_]: δ/ppm = 8.88 (d, *J* = 4.0 Hz, 2H), 8.73 (s, 2H), 7.59 (d*, J* = 4.0 Hz, 2H). ^13^C NMR [150 MHz, CDCl_3_]: δ/ppm = 156.2 (2×Cq), 150.4 (2×CH), 139.9 (2×Cq (CF_3_), *J*_C-F_ = 34.2 Hz), 122.9 (2×Cq (CF_3_), *J*_C-F_ = 273.2 Hz), 120.0 (2×CH (CF_3_), *J*_C-F_ = 3.6 Hz), 117.2 (2×CH (CF_3_), *J*_C-F_ = 3.7 Hz).

Ligand **c** [2,2'-bipyridine]-4,4'-dicarbonitrile ^1^H NMR [600 MHz, CDCl_3_]: δ/ppm = 8.85 (d, *J* = 5.2 Hz, 2H), 8.69 (s, 2H), 7.58 (d, *J* = 4.8 Hz, 2H). ^13^C NMR [150 MHz, CDCl_3_]: δ/ppm = 155.4 (2×Cq), 150.3 (2×CH), 125.8 (2×CH), 123.1 (2×Cq), 121.82 (2×CH), 116.32 (2×Cq).

(E)-3-(2-hydroxyphenyl)-1-phenyl-2-propen-1-one ^1^H-NMR: [400 MHz, CDCl_3_]: δ/ppm = 8.13 (d, *J* = 15,92 Hz, 1H), 8.03 (d, *J* = 7,1, 1H), 7.70 (d, *J* = 15,85, 1H), 7.58 (t, *J* = 8,82, 2H), 7.50 (t, *J* = 7,54, 2H), 7.27 (t, *J* = 7,95, 1H), 6.97 (t, *J* = 7,58, 1H), 6.88 (d, *J* = 8,09, 1H).

Ligand **d** *N*,*N*-dimethyl-4'-(trifluoromethyl)-[2,2'-bipyridin]-4-amine ^1^H NMR (400 MHz, CDCl_3_): δ/ppm = 8.80 (d, *J* = 5.0 Hz, 1H), 8.66 (s, 1H), 8.33 (d, *J* = 5.9 Hz, 1H), 7.72 (d, *J* = 2.7 Hz, 1H), 7.48 (d, *J* = 5.0 Hz, 1H), 6.56 (dd, *J* = 5.9, 2.7 Hz, 1H), 3.10 (s, 6H).^13^C NMR [100 MHz, CDCl_3_]: δ/ppm = 158.4 (Cq), 155.4 (Cq), 154.7 (Cq), 149.8 (CH), 149.5 (CH), 139.2 (Cq, (CF_3_), *J*_C-F_ = 33.7 Hz), 123.1 (Cq (CF_3_), *J*_C-F_ = 273.5 Hz), 118.8 (CH, (CF_3_), *J* = 3.1 Hz), 117.1 (CH, (CF_3_), *J* = 3.1 Hz), 107.3 (CH), 104.2 (CH), 39.4 (2×CH_3_). ^19^F (564 MHz, CDCl_3_): -64.60 (m, 3F). GC-MS: 267 (molecular ion), 252 (base peak), 224. IR (FT-ATR, cm^–1^): 2954, 2925, 2854, 1595, 1478, 1427, 1333, 1161, 990.

Ligand **e** 4,6-diphenyl-2,2'-bipyridine (dpbpy) ^1^H-NMR: [400 MHz, (CD_3_)_2_SO]: δ/ppm = 8.77(d, 4.69 Hz, 1H), 8.65 (d, 7.61 Hz, 1H), 8.63 (s, 1H), 8.34 (d, 7.32 Hz, 2H), 8.32 (s, 1H), 8.05 (d, 7.91 Hz, 1H), 8.00 (d, 7.32 Hz, 2H), 7.5-7.65 (m, 7H). ^13^C-NMR: [100 MHz, (CD_3_)_2_SO]: δ/ppm = 157.11, 156.27, 155.78, 150.19, 149.89, 139.10, 138.22, 138.10, 130.11, 130.00, 129.85, 129.38, 127.74, 127.60, 125.17, 125.05, 121.51, 118.69, 117.13.

Ligand **f** 4-(2-hydroxy-phenyl)-6-phenyl-2,2'-bipyridine (hpbpy) ^1^H-NMR: [400 MHz, (CD_3_)_2_SO]: δ/ppm = 8.73 (d, *J* = 4,81 Hz, 1H), 8.64 (s, 1H), 8.62 (d, *J* = 3,11 Hz, 1H), 8.27 (d, 7,24 Hz, 2H), 8.16 (s, 1H), 8.01 (td, *^1^J* = 8,63 Hz, *^2^J* = 1,8 Hz, 1H), 7.53 (m, 5H), 7.30 (t, *J* = 8,59 Hz, 1H), 7.06 (d, *J* = 9,29 Hz, 1H), 6.98 (t, *J* = 7,45 Hz, 1H). ^13^C-NMR: [100 MHz, (CD_3_)_2_SO]: 172.97, 156.20, 156.10, 155.58, 155.37, 149.85, 148.96, 139.41, 137.93, 130.82, 129.71, 129.39, 127.38, 125.72, 124.79, 121.36, 121.06, 120.20, 119.99, 117.04.

**NMR assignments of complexes and ESI-MS data**

Mn^I^(bpy-4,4’-CF_3_)(CO)_3_Br (**1a**). ^1^H-NMR: [400 MHz, (CD_3_)_2_CO]: δ/ppm = 9.63 (d, *J* = 5.13 Hz, 2H), 9.22 (s, 2H), 8.12 (d, *J* = 4.83 Hz, 2H). ^13^C-NMR: [100 MHz, (CD_3_)_2_CO]: δ/ppm = 156.59, 155.37, 140.14 (q, CF_3_), 123.96, 122.69, 121.23, 120.26. IR-ATR (CO selected bands): ν/cm^–1^ = 2026, 1945, 1918. MS (ESI^–^): m/z = calcd for C_15_H_6_BrF_6_MnN_2_O_3_[M]^–^ 509.88-511.88, found 509.90-511.98.

Mn^I^(bpy-5,5’-CF_3_)(CO)_3_Br (**1b**). ^1^H-NMR: [400 MHz, (CD_3_)_2_CO]: δ/ppm = 9.64 (s, 2H), 9.00 (d, *J* = 6.15 Hz, 2H), 8.70 (d, *J* = 4.83 Hz, 2H). ^13^C-NMR: [100 MHz, (CD_3_)_2_CO]: δ/ppm = 158.12, 150.35, 137.00, 128.72 (q, CF_3_), 125.04, 124.08, 121.37. IR-ATR (CO selected bands): ν/cm^–1^ = 2024, 1941, 1915. MS (ESI^–^): m/z = calcd for C_15_H_6_BrF_6_MnN_2_O_3_[M]^–^ 509.88-511.88, found 509.92-512.01.

Mn^I^(bpy-4,4’-CN)(CO)_3_Br (**1c**) ^1^H-NMR: [400 MHz, (CD_3_)_2_CO]: δ/ppm = 9.60 (d, *J* = 4.83 Hz, 2H), 9.15 (s, 2H), 8.17 (d, *J* = 5.05 Hz). ^13^C-NMR: [100 MHz, (CD_3_)_2_CO]: δ/ppm = 155.95, 155.02, 128.43, 126.10, 122.75, 115.45. IR-ATR (CO selected bands): ν/cm^–1^ = 2030, 1926. Decomposition of the complex, as mentioned in the text, prevented to obtain reproducible MS spectra.

Mn^I^(bpy-4,4’-CF_3_-NMe_2_)(CO)_3_Br (**1d**) ^1^H-NMR: [400 MHz, (CD_3_)_2_CO]: δ/ppm = 9.52 (d, *J* = 5.56 Hz, 1H), 8.86 (s, 1H), 8.65 (d, *J* = 6.44 Hz, 1H), 7.97 (d, *J* = 4.98 Hz, 2H), 6.95 (d, *J* = 6.15 Hz, 1H), 3.24 (s, 6H). ^13^C-NMR: [100 MHz, (CD_3_)_2_CO]: δ/ppm = 159.64, 158.75, 154.94, 153.95, 152.17, 139.64 (q, CF_3_), 121.35, 118.32, 109.64, 106.82, 39.00. IR-ATR (CO selected bands): ν/cm^–1^ = 2022, 1931, 1904. MS (ESI^+^): m/z = calcd for C_18_H_15_F_3_MnN_4_O_3_ [M-Br+MeCN]^+^447.05, found 446.56.

Mn^I^(dpbpy)(CO)_3_Br (**1e**). ^1^H-NMR: [400 MHz, (CD_3_)_2_CO]: δ/ppm = 9.32 (d, *J* = 5.27 Hz, 1H), 8.87 (d, *J* = 7.03 Hz, 2H), 8.25 (t, 1H), 8.11 (d, *J* = 7.61 Hz, 2H), 7.97 (s, 1H), 7.72–7.74 (m, 3H), 7.59-7.61 (m, 6H). ^13^C-NMR: [100 MHz, (CD_3_)_2_CO]: δ/ppm = 165.65, 157.69, 157.32, 153.26, 150.43, 142.77, 138.83, 135.96, 130.48, 129.87, 129.44, 127.69, 126.11, 124.69, 124.06, 119.15. IR-ATR (CO selected bands): ν/cm^–1^ = 2022, 1954, 1925. MS (ESI^+^): m/z = calcd for C_27_H_19_MnN_3_O_3_ [M-Br+MeCN]^+^488.08, found 488.64.

Mn^I^(hpbpy)(CO)_3_Br (**1f**). ^1^H-NMR: [400 MHz, (CD_3_)_2_CO]: δ/ppm = 9.31 (d, *J* = 5.27 Hz, 1H), 9.21 (s, OH), 8.81 (s, 1H), 8.70 (d, *J* = 8.20 Hz, 1H), 8.23 (t, 1H), 7.97 (s, 1H), 7.70-7.73 (m, 4H), 7.58-7.61 (m, 3H), 7.37 (t, 1H), 7.03-7.11 (m, 2H). ^13^C-NMR: [100 MHz, (CD_3_)_2_CO]: δ/ppm= 164.82, 157.74, 156.34, 153.23, 149.05, 142.97, 138.85, 131.49, 130.69, 129.73, 129.44, 127.32, 125.96, 123.68, 121.54, 120.53, 116.79. IR-ATR (CO and OH selected bands): ν/cm^–1^ = 3267, 2022, 1934, 1917. MS (ESI^–^): m/z = calcd for C_25_H_15_BrMnN_2_O_4_^–^ [M-H]^–^ 540.96-542.96, found 541.57-543.59.

Mn^I^(bpy-4,4’-NMe_2_)(CO)_3_Br (**1g**). ^1^H-NMR: [400 MHz, (CD_3_)_2_CO]: δ/ppm = 8.59 (d, *J* = 6.44 Hz, 2H), 7.57 (s, 2H), 6.83 (d, *J* = 6.74 Hz, 2H), 3.19 (s, 12H). ^13^C-NMR: [100 MHz, (CD_3_)_2_CO]: δ/ppm = 156.16, 155.44, 151.98, 108.68, 104.61, 38.85. IR-ATR (CO selected bands): ν/cm^–1^ = 2008, 1914, 1891. MS (ESI^+^): m/z = calcd for C_19_H_21_MnN_5_O_3_ [M-Br+MeCN]^+^422.10, found 422.53.

Re^I^(bpy-4,4’-CF_3_)(CO)_3_Cl (**2a**). ^1^H-NMR: [400 MHz, (CD_3_)_2_SO]: δ/ppm = 9.48 (s, 2H), 9.34 (d, *J* = 5.86 Hz, 2H), 8.17 (d, *J* = 5.13 Hz, 2H). ^13^C-NMR: [100 MHz, (CD_3_)_2_SO]: δ/ppm = 197.73, 189.41, 156.79, 155.24, 140.14 (q, CF_3_), 124.79, 124.08, 122.47, 121.39. IR-ATR (CO selected bands): ν/cm^–1^ = 2019, 1909, 1872. MS (ESI^+^): m/z = calcd for C_17_H_9_F_6_N_3_O_3_Re [M-Cl+MeCN]^+^ 604.01-602.01, found 603.70-601.74.

Re^I^(bpy-5,5’-CF_3_)(CO)_3_Cl (**2b**). ^1^H-NMR: [400 MHz, (CD_3_)_2_SO]: δ/ppm = 9.32 (s, 2H), 9.18 (d, *J* = 8.64 Hz, 2H), 8.93 (d, *J* = 8.49 Hz, 2H). ^13^C-NMR: [100 MHz, (CD_3_)_2_SO]: δ/ppm = 197.61, 189.48, 158.21, 150.14, 138.81, 129.19 (q, CF_3_), 126.82, 124.03, 121.31. IR-ATR (CO selected bands): ν/cm^–1^ = 2023, 1923, 1895. MS (ESI^+^): m/z = calcd for C_17_H_9_F_6_N_3_O_3_Re [M-Cl+MeCN]^+^ 604.01-602.01, found 603.82-601.80.

Re^I^(bpy-4,4’-CN)(CO)_3_Cl (**2c**). ^1^H-NMR: [400 MHz, (CD_3_)_2_SO]: δ/ppm =9.38 (s, 2H), 9.29 (d, *J* = 5.56 Hz, 2H), 8.22 (d, *J* = 4.10 Hz, 2H). ^13^C-NMR: [100 MHz, (CD_3_)_2_SO]: δ/ppm = 197.45, 189.16. 155.89, 154.78, 130.61, 128.07, 123.38, 116.19. IR-ATR (CO selected bands): ν/cm^–1^ = 2019, 1920, 1893. MS (ESI^+^): m/z = calcd for C_17_H_9_N_5_O_3_Re [M-Cl+MeCN]^+^ 518.03-516.02, found 517.71-515.82.

Re^I^(bpy-4,4’-CF_3_-NMe_2_)(CO)_3_Cl (**2d**). ^1^H-NMR: [400 MHz, (CD_3_)_2_SO]: δ/ppm = 9.21 (d, *J* = 5.27 Hz, 2H), 8.37 (d, *J* = 6.74 Hz, 1H), 8.03 (d, *J* = 7.03 Hz,1H), 7.93 (s, 1H), 6.89 (d, *J* = 4.10 Hz, 1H), 3.21 (s, 6H). ^13^C-NMR: [100 MHz, (CD_3_)_2_SO]: δ/ppm = 198.86, 198.30, 191.21, 158.93, 155.65, 154.87, 154.01, 152.06, 123.34, 120.60, 110.05, 107.95. IR-ATR (CO selected bands): ν/cm^–1^ = 2013, 1902, 1880. MS (ESI^+^): m/z = calcd for C_18_H_15_F_3_N_4_O_3_Re [M-Cl+MeCN]^+^ 579.07-577.06, found 578.68-576.67

Re^I^(dpbpy)(CO)_3_Cl (**2e**). ^1^H-NMR: [400 MHz, (CD_3_)_2_SO]: δ/ppm = 9.13 (d, *J* = 7.32 Hz, 1 H), 9.12 (s, 1H), 9.08 (d, *J* = 5.27 Hz, 1H), 8.81 (d, *J* = 4.83 Hz, 1H), 8.40 (t, *J* = 7.91 Hz, 1H), 8.21 (m, 3H), 8.06 (t, *J* = 8.49 Hz, 1H), 7.92 (d, *J* = 7.61 Hz, 1H), 7.79 (t, *J* = 6.44 Hz, 1H), 7.62-7.66 (m, 5H). ^13^C-NMR: [100 MHz, (CD_3_)_2_SO]: δ/ppm = 162.14, 156.85, 153.29, 151.24, 149.81, 140.54, 137.49, 131.51, 129.94, 128.48, 128.06, 126.10, 125.76, 125.52, 124.99, 121.29, 110.84. IR-ATR (CO selected bands): ν/cm^–1^ = 2013, 1903, 1871. MS (ESI^+^): m/z = calcd for C_25_H_16_N_2_O_3_Re [M-Cl]^+^ 579.07-577.07, found 579.83-577.82.

Re^I^(hpbpy)(CO)_3_Cl (**2f**). ^1^H-NMR: [400 MHz, (CD_3_)_2_SO]: δ/ppm = 10.35 (s, 1H), 9.05 (d, *J* = 5,72 Hz, 1H), 8.92 (d, *J* = 1,73 Hz, 1H), 8.90 (s, 1H), 8.35 (td, *^1^J* = 7,84 Hz, *^2^J* = 1,57 Hz, 1H), 8.03 (d, *J* = 1,73 Hz, 1H), 7.77 (m, 2H), 7.61 (m, 5H), 7.38 (td, *^1^J* = 8,63, *^2^J* = 1,65, 1H), 7.04 (m, 2H). ^13^C-NMR: [100 MHz, (CD_3_)_2_SO]: 162.90, 157.25, 156.53, 156.08, 153.28, 150.17, 142.57, 140.53, 132.37, 131.40, 130.38, 129.67, 129.47, 128.77, 127.95, 127.78, 125.70, 123.00, 120.47, 117.23. IR-ATR (CO and OH selected bands): ν/cm^–1^ = 3309, 2020, 1912, 1883. MS (ESI^–^): m/z = calcd for C_25_H_16_ClN_2_O_4_Re [M-H]^–^630.04-628.03, found 629.55-628.14.

Re^I^(bpy-4,4’-NMe_2_)(CO)_3_Cl (**2g**). ^1^H-NMR: [400 MHz, (CD_3_)_2_SO]: δ/ppm =8.30 (d, *J* = 6.74 Hz, 2H), 7.59 (s, 2H), 6.80 (d, *J* = 4.03 Hz, 2H), 3.35 (s, 12 H). ^13^C-NMR: [100 MHz, (CD_3_)_2_SO]: δ/ppm = 199.73, 192.84, 156.28, 155.53, 151.83, 109.30, 105.74. IR-ATR (CO selected bands): ν/cm^–1^ = 2006, 1895, 1865. MS (ESI^+^): m/z = calcd for C_19_H_21_N_5_O_3_Re [M-Cl+MeCN]^+^ 554.12-552.12, found 553.63- 551.61.

**REFERENCES**

Hirshfeld, F. L. (1977). Bonded-atom fragments for describing molecular charge densities. *Theoret. Chim. Acta* 44, 129–138. doi:10.1007/BF00549096.

McKinnon, J. J., Jayatilaka, D., and Spackman, M. A. (2007). Towards quantitative analysis of intermolecular interactions with Hirshfeld surfaces. *Chem. Commun.* 0, 3814–3816. doi:10.1039/B704980C.

McKinnon, J. J., Spackman, M. A., and Mitchell, A. S. (2004). Novel tools for visualizing and exploring intermolecular interactions in molecular crystals. *Acta Crystallogr., B* 60, 627–668. doi:10.1107/S0108768104020300.
